# Extracellular vesicle‐packaged mitochondrial disturbing miRNA exacerbates cardiac injury during acute myocardial infarction

**DOI:** 10.1002/ctm2.779

**Published:** 2022-04-22

**Authors:** Ping Sun, Chao Wang, Ge Mang, Xiangli Xu, Shuai Fu, Jianfeng Chen, Xiaoqi Wang, Weiwei Wang, Hairu Li, Peng Zhao, Yifei Li, Qi Chen, Naixin Wang, Zhonghua Tong, Xin Fu, Ying Lang, Shasha Duan, Dongmei Liu, Maomao Zhang, Jiawei Tian

**Affiliations:** ^1^ Department of Ultrasound The Second Affiliated Hospital of Harbin Medical University Harbin China; ^2^ The Key Laboratory of Myocardial Ischemia Harbin Medical University, Ministry of Education Harbin China; ^3^ Department of Cardiology The Second Affiliated Hospital of Harbin Medical University Harbin China; ^4^ Department of Ultrasound The Second Hospital of Harbin city Harbin China; ^5^ Laboratory Animal Center the Second Affiliated Hospital of Harbin Medical University Harbin China

**Keywords:** extracellular vesicles, miRNA, myocardial infarction, N6‐methyladenosine

## Abstract

Mounting evidence suggests that extracellular vesicles (EVs) are effective communicators in biological signalling in cardiac physiology and pathology. However, the role of EVs in cardiac injury, particularly in ischemic myocardial scenarios, has not been fully elucidated. Here, we report that acute myocardial infarction (AMI)‐induced EVs can impair cardiomyocyte survival and exacerbate cardiac injury. EV‐encapsulated miR‐503, which is enriched during the early phase of AMI, is a critical molecule that mediates myocardial injury. Functional studies revealed that miR‐503 promoted cardiomyocyte death by directly binding to peroxisome proliferator‐activated receptor gamma coactivator‐1β (PGC‐1β) and a mitochondrial deacetylase, sirtuin 3 (SIRT3), thereby triggering mitochondrial metabolic dysfunction and cardiomyocyte death. Mechanistically, we identified endothelial cells as the primary source of miR‐503 in EVs after AMI. Hypoxia induced rapid H3K4 methylation of the promoter of the *methyltransferase‐like 3* gene (*METTL3*) and resulted in its overexpression. METTL3 overexpression evokes N6‐methyladenosine (m^6^A)‐dependent miR‐503 biogenesis in endothelial cells. In summary, this study highlights a novel endogenous mechanism wherein EVs aggravate myocardial injury during the onset of AMI via endothelial cell‐secreted miR‐503 shuttling.

## Graphical abstract

1. Extracellular vesicles serve as early master regulators to exacerbate myocardial injury in acute myocardial infarction (AMI).
2. Exosomal miR‐503 is a critical molecule that triggers mitochondrial dysfunction and cardiomyocyte death via the PGC‐1β/NRF‐1 and SIRT3/PDH/ATP synthase communication axes.
3. The initiation and biogenesis of miR‐503 are dependent on H3K4me3/METTL3‐mediated m^6^A modification of pri‐miR‐503 in cardiac endothelial cells during AMI.

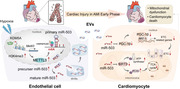

## 1 BACKGROUND

Acute myocardial infarction (AMI), which manifests as ischemia and irreversible death of cardiomyocytes, is the most severe clinical manifestation of ischemic heart disease.^1^ Despite improved reperfusion strategies, evidence‐based therapies, and healthcare logistics, patients undergoing extensive tissue injury during the acute phase exhibit high mortality and can further develop heart failure.^2^ The extent of cardiomyocyte loss in the early stage of AMI is directly associated with subsequent prognosis. In the progression of AMI pathogenesis, mitochondrial dysfunction and energy depletion occur early during cardiac injury. Mitochondrial metabolic dysfunction severely jeopardises cardiomyocyte survival.^3^, ^4^ However, the mechanism disturbing cardiomyocyte mitochondrial homeostasis and cardiomyocyte loss during AMI remains obscure.

Under the stress conditions of AMI, intercellular communications among different cardiac cell populations are widely activated. Recently, there has been an exponential increase in the number of studies that have highlighted extracellular vesicles (EVs) as effective communicators of biological signalling in cardiac pathology processes.^5^ Intercellular communication mediated by EVs following AMI is associated with mitochondrial dysfunction, cardiomyocyte loss, and subsequent progression of heart failure, providing new insights into subsequent research and therapy.^6^, ^7^ Although the crosstalk mediated by EVs has been described in the intracardiac region and long‐distance organs, the biological regulatory effects of EVs on myocardial injury after AMI remain elusive.^7^

EVs mediate message delivery by encapsulating functional molecules. EVs containing miRNAs, whose levels markedly vary during pathological processes, can exert significant effects by regulating gene expression at the posttranscriptional level.^8^ Notably, certain miRNAs prefer to be selectively packed in EVs.^9^ The composition of miRNAs carried by EVs that specifically change after AMI and function as participants in the pathological process remains unknown. The initiation and biogenesis processes of these miRNAs are worth exploring.

In this study, we determine whether AMI‐secreted EVs are critical mediators in sustaining and amplifying cardiac injury signals. Furthermore, we provided the first evidence that EVs containing miR‐503 are specifically expressed in AMI and are related to cardiomyocyte loss. Our results provide insights into the mechanism by which miR‐503 is initiated and its influence on cardiomyocytes. Strategies blocking miR‐503 biogenesis or the administration of miR‐503 antagonists may be leveraged as cardioprotective strategies after AMI.

## 2 MATERIALS AND METHODS

### 2.1 Study design

This study aimed to explore the role of EVs in myocardial injury during the early phase of AMI. We collected EVs from patients with AMI and incubated them with human cardiomyocytes to measure cellular survival. We isolated EVs from the AMI mouse model and injected them into the myocardium to measure the extent of cardiac injury. Using deep sequencing, we assessed exosomal miRNA profiles after AMI and identified the critical molecule (miR‐503) responsible for myocardial injury. Using functional assays, we explored the mechanism by which exosomal miR‐503 was initiated and influenced cardiomyocytes. The experimental methods are described in detail in the Supporting Information.

### 2.2 Subjects and sample collection

Patients with AMI undergoing primary percutaneous coronary intervention for ST‐elevation myocardial infarction (STEMI) were included in the study. STEMI was diagnosed according to the current European Guidelines on AMI.^10^ Patients with stable angina who underwent elective diagnostic and/or interventional coronary procedures were included in the control groups. Stable angina was diagnosed based on the European Society of Cardiology guidelines for stable coronary artery disease.^11^ Patients with pregnancy, tumours, hepatic or nephritic diseases, connective tissue diseases, inflammatory arthritis, systemic infections within the past 3 weeks, previous myocardial infarction (MI), and coronary artery bypass grafting history were excluded. Peripheral blood samples were collected before percutaneous coronary intervention with K_2_EDTA anticoagulant tubes. For functional studies, EVs were isolated from AMI patients in the acute phase within 6 hours. Informed consent was obtained from all enrolled patients. The study was approved by the ethics committee of the Second Affiliated Hospital of Harbin Medical University. The baseline data of the included patients are listed in Table 1.

**TABLE 1:** Baseline clinical characteristics in acute myocardial infarction (AMI) patients and stable angina control group

	**AMI (*n* = 45)**	**Control (*n* = 30)**	***P* value**
Demographics			
Age, yrs	55±14	52±11	0.2372
Men	37 (82.2%)	21 (70.0%)	0.2651
Current smoker	31 (68.8%)	19 (63.3%)	0.6270
Blood pressure, mm Hg			
Systolic	134.5±23.41	127.0±24.12	0.1835
Diastolic	89.84±10.96	86.70±11.01	0.2283
Laboratory data			
Glucose, mmol/l	7.33±2.76	7.49±2.82	0.8068
Total cholesterol, mmol/l	4.77±1.10	5.17±1.38	0.1650
Triglyceride, mmol/l	1.62±1.45	1.80±1.30	0.5780
HDL cholesterol, mmol/l	1.13±0.24	1.12±0.24	0.8981
LDL cholesterol, mmol/l	3.24±0.95	3.49±0.21	0.3174

### 2.3 Animals

Male C57BL/6 mice (8 weeks old) were purchased from the Experimental Animal Center of the Second Affiliated Hospital of Harbin Medical University. To generate an AMI mouse model, mice were anesthetised by intraperitoneal injection of sterile pentobarbital sodium (Sigma–Aldrich, St. Louis, MO, USA) at 50 mg/kg body weight. Endotracheal intubation and mechanical ventilation were then performed. We then performed a left intercostal thoracotomy to expose the heart. The AMI model was established by permanent ligation of the left anterior descending branch 1.5 mm below the margin of the left auricle, as previously described.^12^ The animals were randomly assigned to treatment groups. All animal experiments were approved by the Research Ethics Committee of the Second Affiliated Hospital of Harbin Medical University.

### 2.4 Extracellular vesicle isolation and characterisation

For cell supernatant EV isolation, we first transplanted an equal number of mouse arterial endothelial cells (MAECs) into 10‐cm cell culture plates and changed the conditioned medium containing 10% EV‐depleted foetal bovine serum (FBS) (Vivacell, Shanghai, China). The conditioned medium was collected and centrifuged at 300 g for 10 minutes, followed by 2000 g for 20 minutes at 4°C to remove cellular debris. Next, the supernatant was filtered through 0.22‐μm filters. EVs were pelleted by ultracentrifugation at 100,000 g for 90 minutes. They were then resuspended in phosphate‐buffered saline (PBS) and recollected by ultracentrifugation again at 100,000 g for 90 minutes.^13^

For serum EV isolation, peripheral blood was stored at room temperature for 20 minutes to allow coagulation. Next, the serum was collected and centrifuged at 1000 × g for 5 minutes followed by 10 000 × g for 10 minutes at 4 ℃ and filtrated through 0.1‐μm filters. EVs were collected by ultracentrifugation at 100,000 g for 1 hour, suspended in PBS, and ultracentrifuged again at 100,000 g for 1 hour.^14^ The isolated pellets were dissolved in a resuspension buffer of snap‐freeze aliquots in liquid nitrogen and stored at –80°C for functional studies.^15^

The EV concentration was measured using a BCA Protein Assay Kit (Beyotime, P0012). For cellular studies, EVs were cultured with recipient cells at a concentration of 25 μg/ml. For EV electron microscopy analysis, the samples were embedded in a mixture of 2% uranyl acetate for 1 minute and observed under a transmission electron microscope (HITACHI‐H7650, Japan). In addition, the size distribution of the EVs was quantified using nanoparticle tracking analysis (Malvern, NanoSight S300). For western blotting analysis, the pellets were resuspended and evaluated for the expression of the exosomal markers Alix (Abcam, ab186429), TSG101 (Abcam, ab125011), and CD63 (Abcam, ab134045) and the exosomal contaminant marker Calnexin (Abcam, ab133615) at an antibody dilution of 1:1000 in a primary antibody dilution buffer (Beyotime, P0023A).

### 2.5 Statistical analysis

All data are reported as the means ± standard deviations. Student's *t*‐test was used to compare the two groups. Multiple groups were compared using one‐way or two‐way analysis of variance (ANOVA) with Bonferroni's post‐hoc correction test. Pearson product‐moment correlation was used to assess the relationship between the parameters. Mouse survival was analysed using the log‐rank test. Statistical significance was set at *P* < 0.05.

## 3 RESULTS

### 3.1 EVs from patients with AMI impair cardiomyocyte survival

**FIGURE 1:**
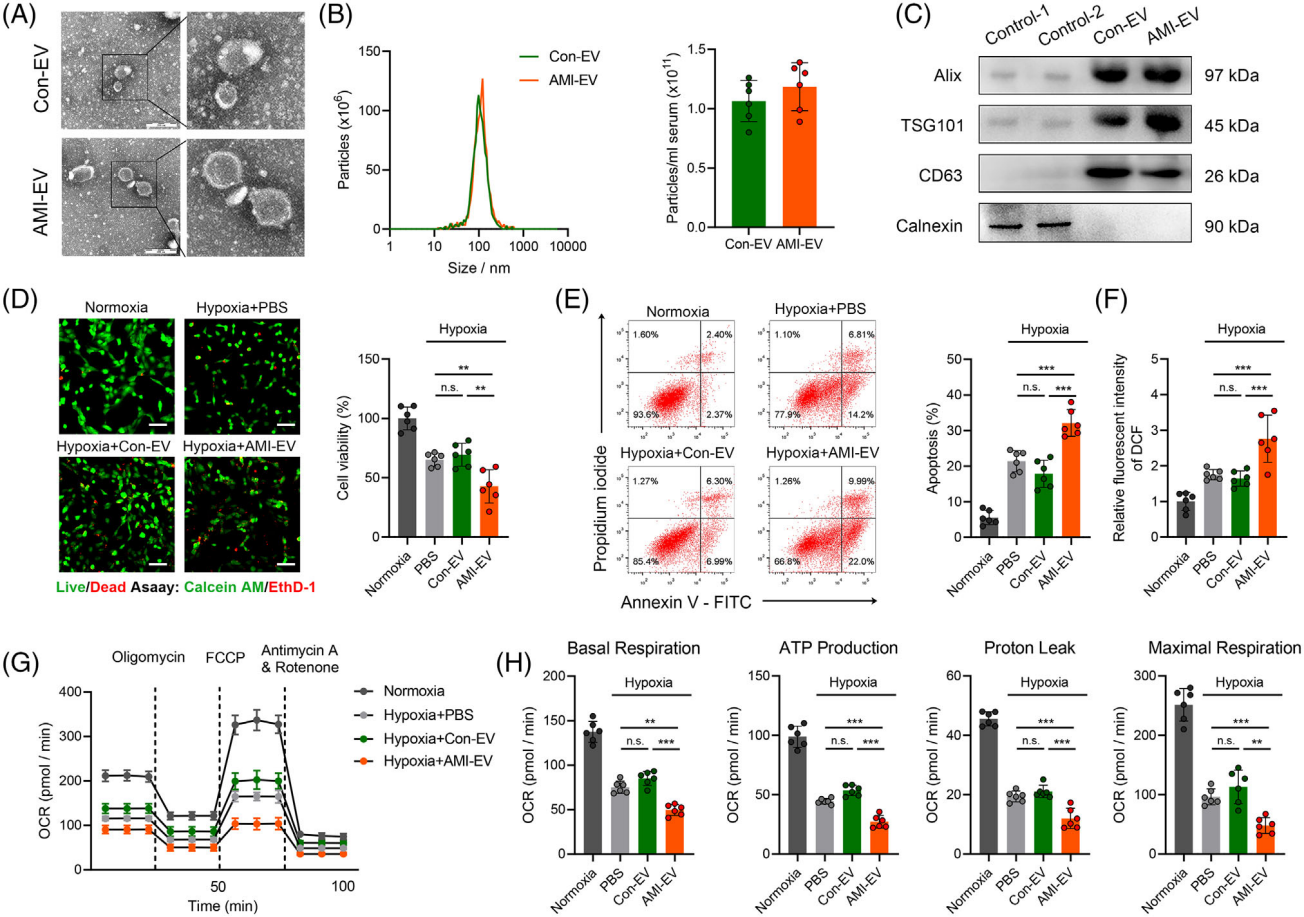
Characterisation and functional validation of acute myocardial infarction (AMI) serum extracellular vesicles (EVs) in human cardiomyocytes AC16. (A) Representative transmission electron micrograph of isolated vesicles from the serum of AMI patients and control patients. Scale bar: 100 nm. (B) Representative images of nanoparticle tracking analysis reflecting the size distributions of isolated vesicles and average concentrations of serum EVs in the AMI and control groups. (C) Representative blot images of exosomal markers (Alix, TSG101, CD63) and the contamination marker calnexin. (D) Fluorescence staining with calcein‐AM (green) vital dyes represents living cells, and ethidium homodimer‐1 staining (red) represents dead cells in the indicated groups. The percentage of cell viability is shown in the right panels. Scale bar: 100 μm. (E) Flow cytometry analysis of Annexin V‐APC and PI fluorescence in AC16 cells incubated with EVs. (F) The effect of EVs on AC16 cell reactive oxygen species (ROS) production was measured using a fluorescence microplate reader to determine the fluorescence intensity of 2,7‐dichlorodihydrofluorescein diacetate (DCF). (G) Cellular oxygen consumption rate (OCR) levels at the indicated times from the basal level following the presence of the indicated drugs. Oligomycin, carbonyl cyanide 4‐(trifluoromethoxy) phenylhydrazone (FCCP), antimycin A, and rotenone. (H) Indices of mitochondrial respiration calculated according to the OCR profiles: basal respiration, adenosine triphosphate (ATP) production, proton leakage, and maximal respiration in AC16 cells. (*n* = 6; **P < 0.01; ***P < 0.001)

Using differential centrifugation, we generated EVs from the serum of patients with AMI (AMI‐EVs) within 6 hours and the control group (Con‐EV). Transmission electron microscopy (TEM) revealed isolated pellets with typical spherical and bilayer‐membrane structures with diameters of approximately 100 nm^16^ (Figure 1A). Using nanoparticle tracking analysis (NTA), we identified the number and size distribution of the vesicles and confirmed no significant differences in the size distribution or serum concentration between the two groups (Figure 1B). The levels of exosomal markers Alix, TSG101, and CD63 were enriched in the isolated EVs but not in the cell lysates. In contrast, calnexin, a mitochondrial protein, was absent in the EVs (Figure 1C). Using laser scanning confocal microscopy, we observed that when Con‐EVs or AMI‐EVs labelled with the fluorescent dye PKH67 were cocultured with AC16 cells, the EVs were internalised by the AC16 cells (Figure S1A). Moreover, AC16 cells exhibited high internalisation efficiency, with > 90% of cells positive for PKH67 fluorescence after 24 hours, as measured using flow cytometry. The uptake efficiency did not differ between the Con‐EV and AMI‐EV groups (Figure S1B).

We then explored the effects of AMI‐EVs on AC16 cell status in response to hypoxia. Notably, the cell viability was decreased by AC16 cell incubation with AMI‐EVs under hypoxia (Figure 1D). The AC16 cells treated with the AMI‐EV‐incubated cells exhibited increased apoptosis compared to the PBS or Con‐EV groups (Figures 1E; Figure S2A). By measuring the mitochondrial membrane potential (ΔΨm), we concluded that AMI‐EVs increased the cellular ΔΨm loss under hypoxia (Figure S2B). Moreover, these pathological EVs promoted reactive oxygen species (ROS) accumulation in AC16 cells (Figure 1F). By measuring the oxygen consumption rate (OCR), we observed AMI‐EV stimulation‐induced OCR reduction compared to the PBS or Con‐EV groups in response to hypoxia, as quantified by the respiration parameters indicating basal respiration, adenosine triphosphate (ATP) production, proton leakage, and maximal respiration (Figure 1G,H). Therefore, these findings indicate that AMI‐EVs are an adverse factor for cardiomyocyte survival and could accelerate cellular death.

### 3.2 Intramyocardial delivery of circulating EVs aggravated cardiac injury after AMI

**FIGURE 2:**
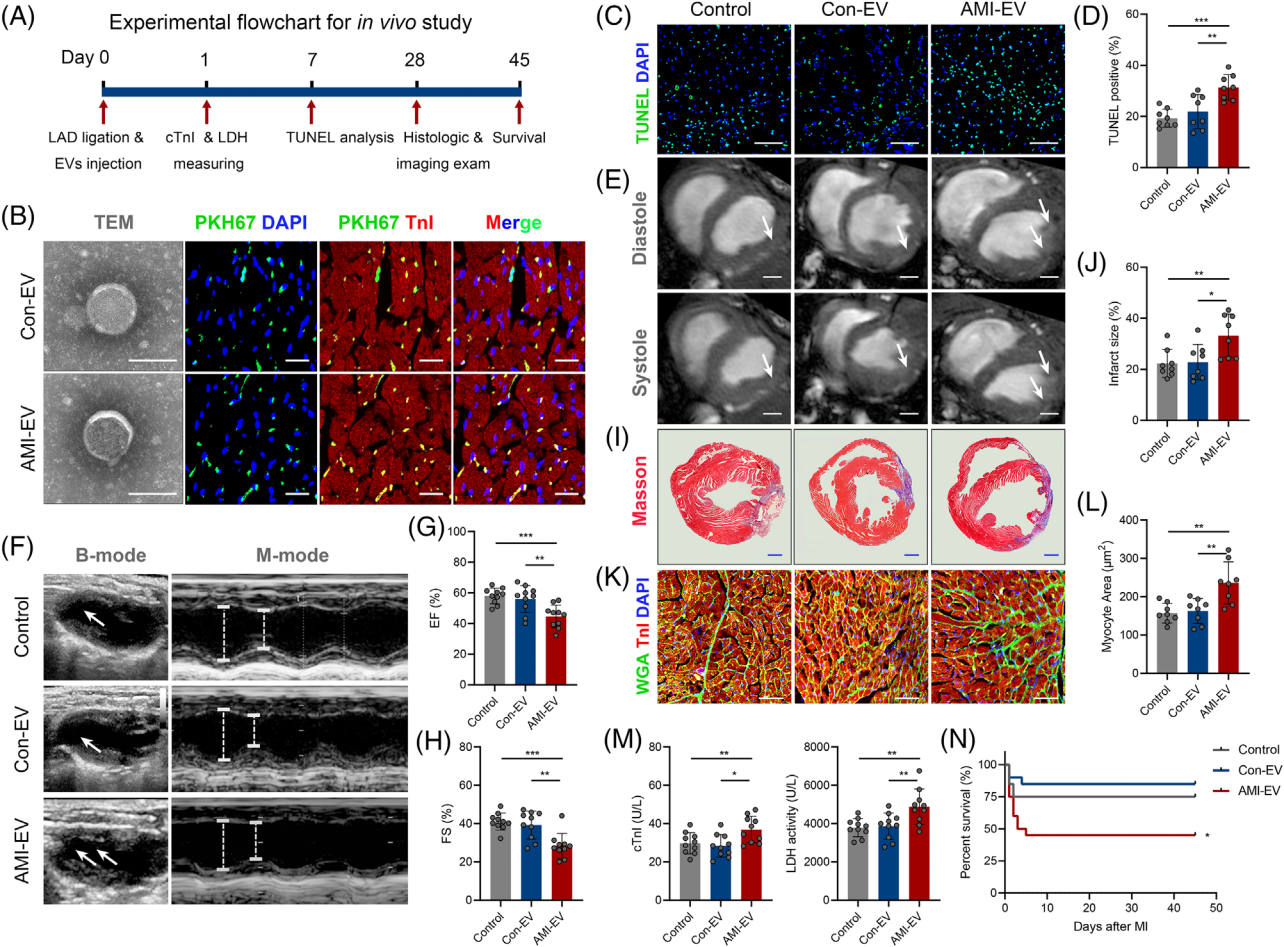
Extracellular vesicles (EVs) isolated from the serum of acute myocardial infarction (AMI) mice with exacerbated myocardial injury and cardiac dysfunction. (A) Flowchart of the in vivo studies. (B) Left, representative transmission electron micrograph of isolated vesicles from the serum of the myocardial infarction (MI) group and sham groups. Scale bar: 100 nm. Right, confocal microscopy images of PKH67‐labelled (green) EVs taken by cTnI+ (red) cardiomyocytes. Scale bar: 100 μm. (C) TUNEL immunofluorescence staining identifies apoptotic cells, followed by DAPI staining to visualise nuclei. Scale bar: 100 μm. (D) Quantification of TUNEL‐positive cardiomyocytes is shown in the right panels (*n* = 8). (E) Representative cardiac magnetic resonance images (MRI) of two‐chamber short‐axis views at the end of diastole and systole. Scale bar: 1 mm. (F) Cardiac function measured by echocardiography 28 days after MI onset. (G, H) The percentage of left ventricular ejection fraction (EF) and fractional shortening (FS) is shown in the right panels (*n* = 10). (I) Representative images of cardiac sections with Masson's trichrome staining at 28 days after MI onset. Scale bar: 1 mm. (J) The quantification of infarct size is shown (*n* = 8). (K) Wheat germ agglutinin (WGA) dye was used for the border zone of the infarct sections to identify the cardiomyocyte size, followed by cTnI immunofluorescence staining for visualising cardiomyocytes and DAPI staining for visualising nuclei. Scale bar: 100 μm. (L) Quantification of the cardiomyocyte size area (*n* = 8). (M) Serum cTnI levels and lactic acid dehydrogenase (LDH) activity at one day after MI onset (*n* = 10). (N) The mouse survival rate was analysed after EV injection (*n* = 20). (*P < 0.05; **P < 0.01; ***P < 0.001)

AMI was experimentally induced by left anterior descending (LAD) ligation, and EVs were isolated from control sham (Con‐EV) and AMI mice (AMI‐EV) to measure their role in myocardial injury after AMI (Figure 2A). First, we determined that there were no significant differences in size distribution and serum concentration between the Con‐EVs and AMI‐EVs from mice (Figure S3A). The levels of exosomal markers TSG101 and CD63 were enriched in EVs isolated from mice (Figure S3B). TEM confirmed that the EVs were ∼100 nm in diameter and had bilayered membranes, which is in accordance with the characteristics of EVs (Figure 2B). To gain insight into the function of EVs on cardiomyocyte survival, we isolated EVs from different phases of LAD ligation in mice and incubated them with neonatal mouse cardiomyocytes (NMCMs). AMI‐EVs showed a cardiomyocyte damage effect, with the most evident effect at 1 hour after AMI (Figure S4A,B). Then, we intramyocardially injected EVs into mice to explore the role of EVs in cardiomyocytes in vivo. Serum lactic acid dehydrogenase (LDH) levels were elevated after the injection of AMI‐EVs, indicating that myocardial injury was induced by these EVs (Figure S5A). Using terminal deoxynucleotidyl transferase dUTP nick end labelling (TUNEL) staining, we observed that the delivery of AMI‐EVs induced cardiomyocyte apoptosis (Figure S5B). Therefore, these data suggest that AMI‐EVs are harmful factors and could induce cardiomyocyte injury.

To explore the role of these EVs in myocardial infarction conditions, we intramyocardially injected Con‐EVs or AMI‐EVs into mice post‐LAD ligation. PKH‐67 staining and cardiomyocyte‐specific (cTnI^+^) immunofluorescent staining indicated that the EVs were colocalized with cTnI^+^ cells 1 hour after injection, indicating efficient uptake of the EVs by cardiomyocytes in vivo (Figure 2B). Notably, the mice receiving AMI‐EVs exhibited more cardiomyocyte apoptosis, as determined by the TUNEL assay, than those receiving PBS or Con‐EVs (Figure 2C,D). We then focused on the effect of EVs on cardiac function after AMI by echocardiography and magnetic resonance imaging. The results revealed no significant difference in the ejection fraction (EF) or fractional shortening (FS) among the groups at baseline (Figure S6A,B). The AMI‐EV treatment exhibited worsening EF and FS compared to the PBS or Con‐EV groups after MI, indicating that AMI‐EV exacerbated left ventricle (LV) dysfunction post‐MI (Figure 2E–H). Using Masson's trichrome staining, we observed that the AMI‐EV‐treated hearts exhibited a pronounced increase in infarct size compared to the Con‐EV and PBS control groups, indicating that AMI‐EV exacerbated LV remodelling after MI (Figure 2I,J). Moreover, the cross‐sectional areas of cardiomyocytes were higher in the AMI‐EV group than in the Con‐EV and PBS control groups, suggesting that cardiac remodelling was accelerated by AMI‐EVs (Figure 2K,L). Concomitantly, we observed that the levels of serum cTnI and LDH were increased by AMI‐EV injection but not by Con‐EV injection (Figure 2M). Furthermore, the survival rate was lower in the AMI‐EV‐treated mice than in the Con‐EV mice (Figure 2N). Overall, these results suggest that EVs are crucial players in myocardial injury after AMI, the sequelae of adverse LV remodelling, and heart failure.

### 3.3 EV‐encapsulated miR‐503 is highly expressed in the early phase of AMI

**FIGURE 3:**
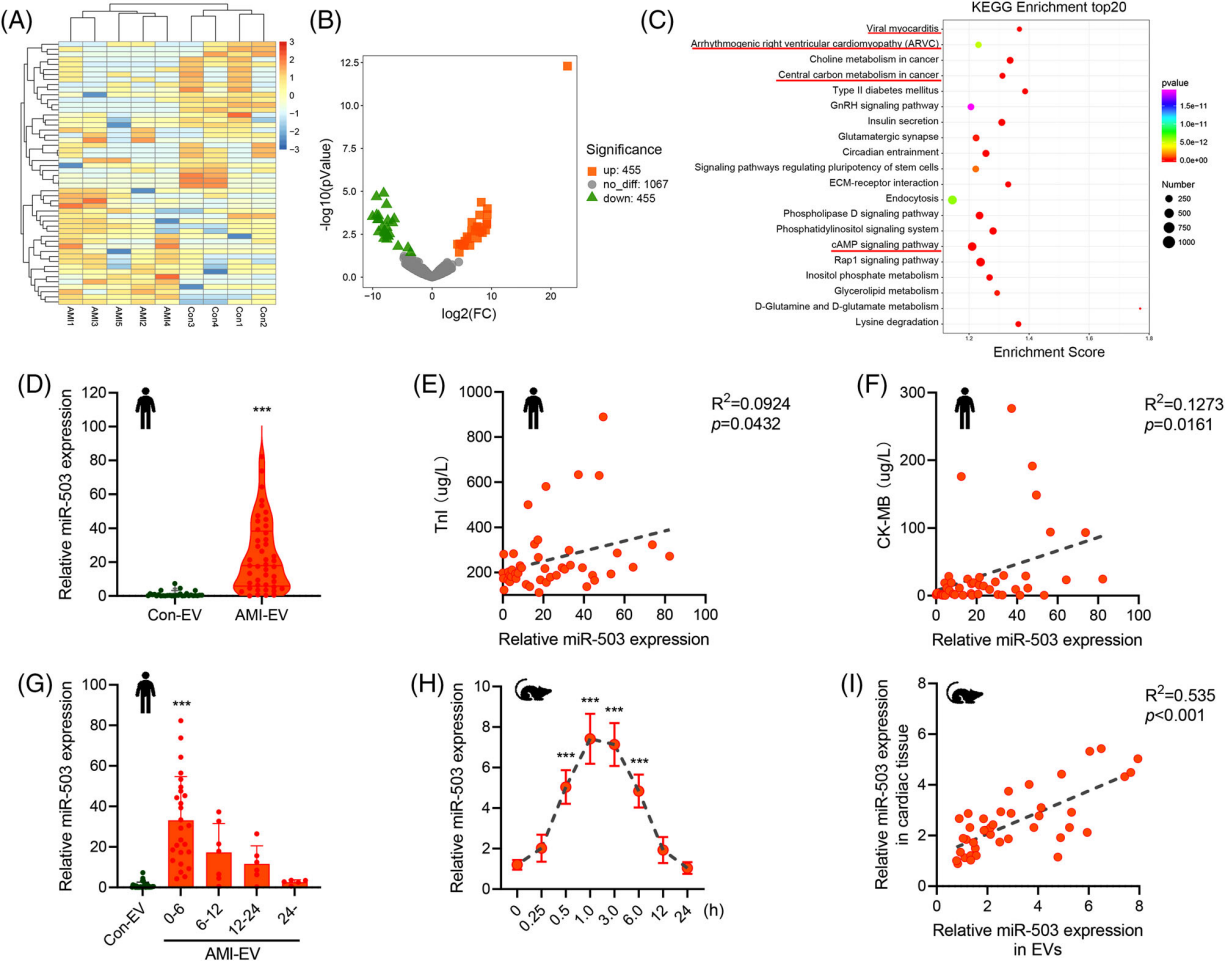
Dysregulation of miR‐503 in acute myocardial infarction (AMI) circulating extracellular vesicles (EVs). (A) Exosomal small RNA sequences from AMI (*n* = 5) and control (*n* = 4) patients are presented in the heatmap. (B) The expression change profiles of exosomal miRNAs from the AMI patients and control groups are presented in the volcano plot. (C) Analysis of the Kyoto Encyclopedia of Genes and Genomes (KEGG) for the predicted target genes of the upregulated miRNAs from small RNA sequences. (D) Real‐time quantitative polymerase chain reaction (qRT–PCR) analysis of miR‐503 expression in serum EVs from the AMI and control groups. (E) Correlation analysis between exosomal miR‐503 expression and serum TnI levels. (F) Correlation analysis between exosomal miR‐503 expression and serum CK‐MB levels. (G) Levels of miR‐503 in the indicated period in post myocardial infarction (MI) patients. (H) qRT–PCR analysis of the levels of miR‐503 in the indicated period in post‐left anterior descending (LAD) ligation mice (*n* = 5). (I) Correlation analysis between exosomal miR‐503 levels and cardiac miR‐503 expression in mice after LAD ligation. (***P < 0.001)

Various signalling molecules carried by EVs are involved in cell‐to‐cell communication. To understand the mechanisms by which the components potentiate myocardial injury, we transfected total or small RNA extracted from AMI‐EVs or Con‐EVs from patients into AC16 cells and measured cell apoptosis under hypoxia. We observed that the transfection of total or small RNA extracted from AMI‐EVs, but not Con‐EVs, recapitulated the acceleration of apoptosis and impaired viability under hypoxia, thereby indicating that the unique small RNA content of AMI‐EVs functions as an apoptosis regulator in cardiomyocytes (Figure S7A,B). To identify the EV‐associated small RNAs that induce cardiomyocyte loss, we selected and profiled the total small RNAs in the EVs by deep sequencing. EVs from AMI‐EVs and Con‐EVs exhibited a similar small RNA biotype distribution. The miRNAs were the top enriched biotypes in AMI‐EV and Con‐EV (47.8% ± 4.0%; 47.3% ± 1.8%) (Figure S7C). Using the threshold cut‐off of two fold change and *P* value < 0.05, we identified 52 miRNAs with significant differences in distribution between the AMI‐EV and Con‐EV groups (Figure 3A,B). We focused on miRNAs that are known for their gene regulatory function and identified a list of differentially secreted miRNAs between the two groups, including 12 upregulated and 11 downregulated miRNAs (Table S1). Next, we performed target gene prediction for the upregulated miRNAs and Kyoto Encyclopedia of Genes and Genomes (KEGG) analyses. We found that the upregulated miRNAs were mainly related to myocardial injury and metabolism pathways, including the central carbon metabolism and cyclic adenosine 3′,5′‐monophosphate (cAMP) signalling pathways (Figure 3C). Then, we evaluated the upregulated miRNA levels in mice 6 hours after AMI and found that the levels of some EV‐associated miRNAs were also increased in mice, including miR‐133a‐3p, miR‐151a‐5p, miR‐199b‐5p, miR‐374b‐5p, miR‐503‐5p, and miR‐708‐5p (Figure S7D). The functions of these upregulated miRNAs on cardiomyocyte survival were evaluated under hypoxia. miR‐503‐5p (also called miR‐503) is a special candidate because of its crucial regulatory role in modulating cell apoptosis and survival (Figure S7E,F). Moreover, the levels of miR‐503 in EVs were unchanged with RNase A treatment but significantly decreased when using a combination of RNase A and Triton X‐100, which demonstrated that the majority of miR‐503 was within EVs (Figure S7G). In addition, depleting EVs from serum through ultracentrifugation almost depleted serum miR‐503 levels (Figure S7H).

Real‐time quantitative polymerase chain reaction (qRT–PCR) analysis confirmed that the exosomal miR‐503 level in the AMI group was dramatically higher than that in the control group (Figure 3D). Specifically, the exosomal miR‐503 level positively correlated with the cardiac injury marker levels, including those of CK‐MB and TnI, in patients with AMI (Figure 3E,F). Moreover, we found that exosomal miR‐503 levels within 6 hours were significantly higher than those at other time points (Figure 3G).

Next, we examined miR‐503 levels in the mice after LAD ligation. We observed that the induction of exosomal miR‐503 expression was sustained throughout the early stage of MI (< 6 hours), with peak expression at 1 hour after ligation and expression returning to baseline after 12 hours (Figure 3H). In addition, exosomal miR‐503 levels positively correlated with miR‐503 expression in cardiac tissue (Figure 3I). Therefore, the level of exosomal miR‐503 was specifically increased after the early phase of MI and was related to myocardial injury.

### 3.4 Exosomal miR‐503 serves as a potent mediator of myocardial injury after AMI

**FIGURE 4:**
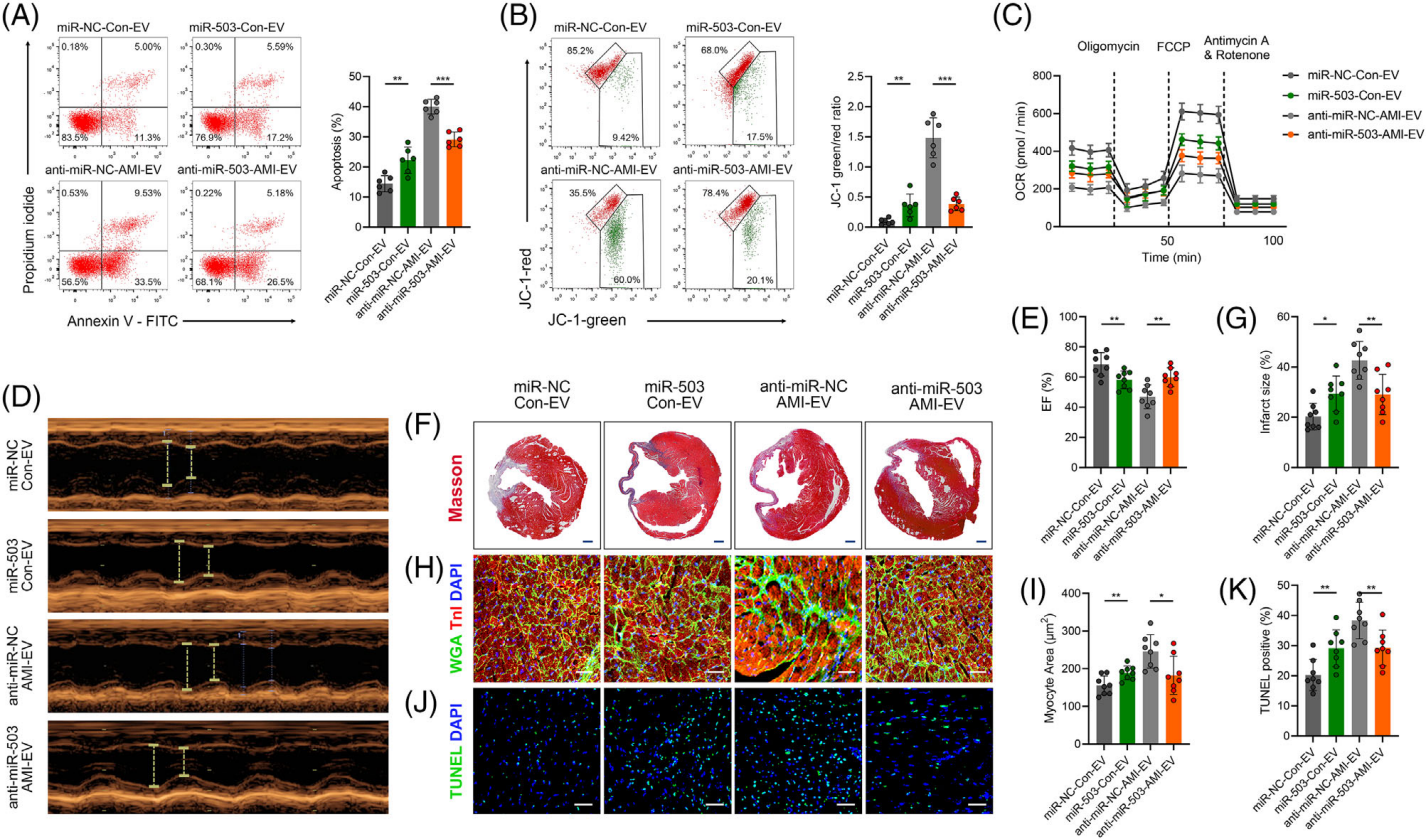
Functional validation of exosomal miR‐503 in cardiomyocytes in vitro and in vivo. (A) Flow cytometry analysis of Annexin V‐APC and PI staining in cardiomyocytes treated with extracellular vesicles (EVs). The percentage of cell apoptosis was calculated and is shown in the right panels. (B) Flow cytometry analysis of mitochondrial membrane depolarisation by JC‐1 staining in cardiomyocytes. (C) Oxygen consumption rate (OCR) changes with time, at the basal level and following the presence of oligomycin, FCCP, antimycin A, and rotenone. (D, E) Cardiac function measured by echocardiography 28 days after myocardial infarction (MI) onset. The percentage of left ventricular ejection fraction (EF) is shown in the right panels. (F, G) Representative images of Masson's trichrome staining of the heart 28 days after MI onset. Scale bar: 1 mm. Quantification of infarct size is shown in the right panels. (H, I) WGA staining to identify the cardiomyocyte size, followed by cTnI immunofluorescence staining and DAPI staining. Scale bar: 100 μm. Cardiomyocyte size area quantification is shown in the right panels. (J, K) TUNEL immunofluorescence staining identifies apoptotic cells, followed by cTnI immunofluorescence staining and DAPI staining. Scale bar: 100 μm. Quantification of TUNEL‐positive cardiomyocytes is shown in the right panels. (*n* = 6 for in vitro studies and *n* = 8 for in vivo studies; *P < 0.05; **P < 0.01; ***P < 0.001)

To determine whether miR‐503 enriched in AMI‐EVs could be transferred to cardiomyocytes, we measured miR‐503 levels in cardiomyocytes treated with AMI‐EVs or Con‐EVs from mice. A considerable increase in the cellular miR‐503 levels was observed in cardiomyocytes following exposure to AMI‐EVs starting from 3 hours of incubation (Figure S8A). The miR‐503 mimic was transfected into Con‐EVs, while an inhibitor of miR‐503 was transfected into AMI‐EVs (Figure S8B). Then, the transected EVs were cocultured with neonatal mouse cardiomyocytes (NMCMs). After 24 hours of incubation, we observed an increase in miR‐503 levels in the cells treated with Con‐EVs transfected with miR‐503 compared to the miRNA negative control group. AMI‐EVs transfected with miR‐503 inhibitor showed decreased miR‐503 levels in cardiomyocytes (Figure S8C). Furthermore, we observed that transfection of Con‐EV with miR‐503 decreased cardiomyocyte viability, increased cell apoptosis and LDH activity, whereas miR‐503‐deficient EVs reversed AMI‐EV‐mediated cardiomyocyte damage (Figure 4A; Figure S9A,B). JC‐1 and DCFHDA staining indicated that exotic miR‐503 induces ΔΨm loss and promotes ROS production, similar to the effect observed in AMI‐EVs. However, AMI‐EVs transfected with the miR‐503 inhibitor effectively reduced this mitochondrial dysfunction (Figure 4B; Figure S9C). Further mitochondrial respiration analysis revealed that exosomal miR‐503 transfection markedly suppressed OCR in cardiomyocyte mitochondria. The miR‐503 inhibitor rescued AMI‐EV‐induced mitochondrial metabolism injury, as measured by basal respiration, ATP production, proton leakage, and maximal respiration (Figure 4C; Figure S9D).

Then we intramyocardially injected Con‐EVs transfected with miR‐503 mimic or AMI‐EVs transfected with a miR‐503 inhibitor into AMI mice. Notably, injection of AMI‐derived miR‐503‐abundant EVs effectively delivered miR‐503 to the myocardium, and the miR‐503 inhibitor obviously blocked the upregulation of miR‐503 expression induced by AMI‐EVs (Figure S8D). Echocardiography indicated that transfection with miR‐503 in Con‐EV induced cardiac dysfunction, while the miR‐503 inhibitor abrogated the AMI‐EV effect on cardiac function (Figure 4D,E). Furthermore, miR‐503‐abundant EVs enlarged the infarct size, whereas miR‐503 inhibition reversed this effect compared to AMI‐EVs transfected with the negative control (Figure 4F,G). In addition, cardiac remodelling and cardiomyocyte apoptosis were induced by miR‐503 but restored by miR‐503 inhibition (Figure 4H‐K). Therefore, exosomal miR‐503 is a crucial mediator of early myocardial injury post MI.

### 3.5 AMI‐EV miR‐503 silences PGC‐1β and SIRT3 to regulate mitochondrial dysfunction

**FIGURE 5:**
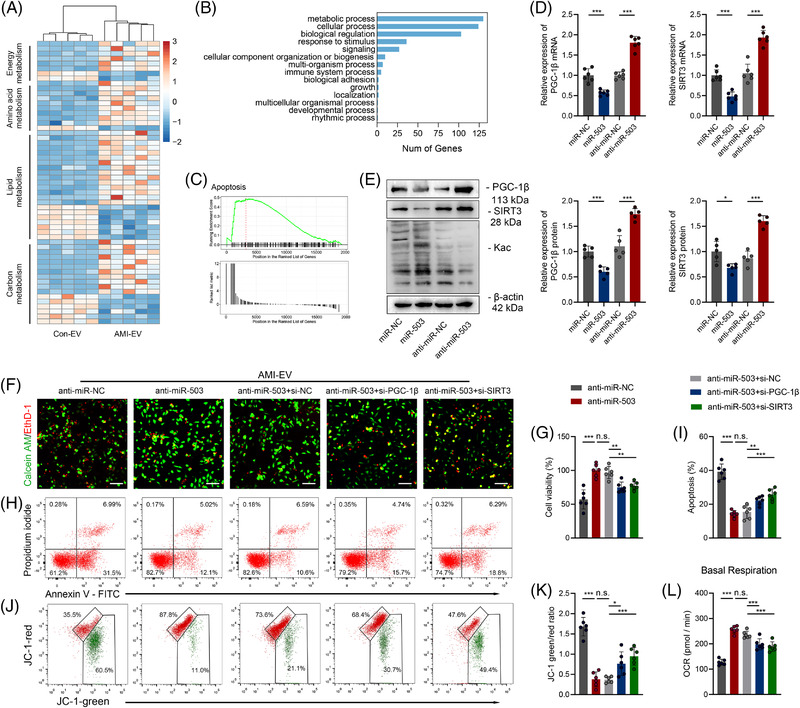
Identification of PGC‐1β and SIRT3 as functional target genes downstream of miR‐503. (A) Heatmap of metabolism‐related differentially expressed genes identified from RNA‐sequencing analysis in mice after left anterior descending (LAD) ligation treated with extracellular vesicles (EVs) from acute myocardial infacrtion (AMI) (AMI‐EV) mice or the control group (Con‐EV). (B) Top 10 KEGG pathways that show differentially expressed genes enriched in cardiac tissue between the Con‐EV and AMI‐EV groups. (C) Gene set enrichment analysis (GSEA) of the apoptosis pathway in the AMI‐EV versus Con‐EV groups. (D) Real‐time quantitative polymerase chain reaction (qRT–PCR) analysis of PGC‐1β and SIRT3 expression in cardiomyocytes transfected with miR NC, miR‐503, anti‐miR‐NC, or anti‐miR‐503. (E) Western blot images reflecting PGC‐1β and SIRT3 protein levels and global acetylation levels in cardiomyocytes transfected with miR‐503 or anti‐miR‐503. (F, G) Calcein‐AM staining (green) and ethidium homodimer‐1 staining (red) for cardiomyocytes transfected with siRNAs and anti‐miR‐503 followed by treatment with AMI‐EVs. Scale bar: 100 μm. (H, I) Flow cytometry analysis of Annexin V‐APC and PI fluorescence in the indicated groups. (J, K) Mitochondrial membrane depolarisation level analysed by JC‐1 staining in the indicated groups. (L) Basal respiration calculated by an XF24 Extracellular Flux Analyser. (*n* = 6; *P < 0.05; **P < 0.01; ***P < 0.001)

Subsequently, we used RNA sequencing to determine the downstream mRNA regulation mechanism and identified several differentially expressed genes involved in metabolism in AMI‐EV‐treated mouse cardiac tissue compared to the Con‐EV group (Figure 5A). Among the top KEGG pathways associated with significantly altered genes between the Con‐EV and AMI‐EV groups, genes involved in metabolic processes were significantly activated (Figure 5B). Further gene set enrichment analysis of the enriched genes indicated that the apoptosis pathway was upregulated in the AMI‐EV group compared to the Con‐EV group (Figure 5C).

We used online databases to predict the miR‐503 target genes. The predicted target genes were then analysed with the downregulated molecules in the cardiac tissue treated with EVs (Figure S10A). The overlapping genes were further validated after miR‐503 transfection (Figure S10B). We focused on peroxisome proliferator‐activated receptor gamma coactivator‐1β (Ppargc‐1β, also called PGC‐1β) and a mitochondrial deacetylase, sirtuin 3 (SIRT3), for their irreplaceable roles in mitochondrial homeostasis and cardiomyocyte survival.^17^, ^18^ Moreover, we found that the PGC‐1β and SIRT3 levels were decreased in LV tissue post ligation (Figure S11A,B). The levels of PGC‐1β and SIRT3 were negatively correlated with those of miR‐503 in cardiac tissue as well as EVs (Figure S11C,D). Furthermore, we observed reduced levels of PGC‐1β and SIRT3 but increased protein acetylation levels in the AMI‐EV group (Figure S11E).

Using 3′ untranslated region (UTR) luciferase reporter assays, we found that miR‐503 mimics significantly inhibited luciferase activity in 293T cells expressing wild‐type *PGC‐1β* and *SIRT3* 3′ UTR reporters, whereas anti‐miR‐503 specifically abolished the suppression. In contrast, mutations in the miR‐503‐binding seed region of the *PGC‐1β* or *SIRT3* 3′ UTR abrogated the repressive effect of miR‐503, validating *PGC‐1β* and *SIRT3* as direct target genes for miR‐503 (Figure S12A,B). AMI‐EV also effectively reduced the luciferase activity in 293T cells expressing wild‐type *PGC‐1β* and *SIRT3* 3′ UTR reporters but not in cells with binding seed region mutations (Figure S12C). In agreement with the above data, the miR‐503 mimic resulted in decreased PGC‐1β and SIRT3 expression at both the mRNA and protein levels, accompanied by increased total protein acetylation. In contrast, miR‐503 inhibition elevated PGC‐1β and SIRT3 expression and decreased acetylation levels (Figure 5D,E). In addition, cardiomyocytes treated with Con‐EV‐overexpressing miR‐503 showed lower PGC‐1β and SIRT3 levels, whereas treatment with AMI‐EV‐deficient miR‐503 increased PGC‐1β and SIRT3 levels (Figure S12D). Furthermore, we transfected cardiomyocytes with biotin‐labelled miR‐503 and demonstrated that *SIRT3* mRNA levels were significantly enriched after RNA pull‐down, indicating the binding of miR‐503 with *PGC‐1β* and *SIRT3* (Figure S12E). Next, we used small interfering RNAs (siRNAs) targeting PGC‐1β and SIRT3 to explore whether exosomal miR‐503 modulated cardiomyocyte death and mitochondrial dysfunction by suppressing the expression of its potential targets PGC‐1β and SIRT3. Notably, we found that miR‐503 inhibition ameliorated cell viability, and mitochondrial homeostasis was abolished by si‐PGC‐1β or si‐SIRT3 (Figure 5F‐L).

PGC‐1 coactivators (PGC‐1s) orchestrate mitochondrial biology to regulate critical metabolic genes for maintaining mitochondrial electron transport chain (ETC) homeostasis. We found that the expression of genes encoding ETC complex I genes (NDUFs), dehydrogenase subunit B (SDHB, complex II), and Fe‐S core protein (Fe‐S, complex III) was significantly decreased in cardiac tissues following AMI (Figure S13A). Moreover, the expression of NDUFs, SDHB, and Fe‐S was downregulated after miR‐503 transfection (Figure S13B). Moreover, PGC‐1s can regulate mitochondrial function by directly coactivating transcription factors, including nuclear respiratory factor 1 (NRF‐1).^19^ We administered si‐NRF‐1 to cardiomyocytes under hypoxia and transfected them with the inhibitor of miR‐503. The results showed that although there were no differences in NRF‐1 expression after the modulation of miR‐503, si‐NFR‐1 could significantly reverse the ETC complex‐related gene upregulation induced by miR‐503 blockade (Figure S13C). Therefore, we concluded that miR‐503‐rich EVs might trigger mitochondrial dysfunction partly through the PGC‐1β/NRF‐1 axis. We then transfected cardiomyocytes with a *PGC‐1β‐* or *SIRT3*‐overexpressing plasmid and cocultured them with Con‐EVs or AMI‐EVs. Consequently, we observed increased PGC‐1β expression as well as ETC complexes I and II levels by transfection with the PGC‐1β plasmid but decreased PGC‐1β, ETC complexes I and II expression following AMI‐EV treatment. Meanwhile, SIRT3‐plasmid‐induced SIRT3 expression accompanied by decreased total protein acetylation was reversed by AMI‐EV treatment (Figure S13D,E).

SIRT3‐induced deacetylation modifications are essential for maintaining mitochondrial function homeostasis by regulating pyruvate dehydrogenase (PDH) and ATP synthase.^20^ Using immunoprecipitation with an antibody against acetylated lysine (Kac), we observed an increase in the acetylation levels of PDH and ATP synthase in response to miR‐503 (Figure S14A). miR‐503 further resulted in a reduction in PDH and ATP synthase activity (Figure S14B). Consistently, *SIRT3* overexpression induced higher PDH and ATP synthase activity, whereas this effect was attenuated by AMI‐EV (Figure S14C,D). Therefore, AMI‐EVs enriched with miR‐503 can induce mitochondrial dysfunction by suppressing SIRT3 expression, thereby boosting the acetylation of key mitochondrial metabolism enzymes.

Furthermore, we performed in vivo rescue experiments to establish the functional link between miR‐503 and its potential target genes. miR‐503 antagomirs, si‐PGC‐1β, and si‐SIRT3 were intramyocardially injected immediately following LAD ligation as previously described.^21^ Notably, we confirmed that the expression of PGC‐1β and SIRT3 was significantly increased by miR‐503 antagomir treatment, whereas the expression was downregulated by siRNA delivery (Figure S15A). Four weeks after LAD ligation, the improved cardiac function and infarct size induced by miR‐503 antagomir injection were exacerbated by treatment with si‐PGC‐1β and si‐SIRT3 (Figure S15B–E). Thus, *PGC‐1β* and *SIRT3* are functional target genes of miR‐503 that exert cardiomyocyte injury effects.

Collectively, these findings demonstrate that AMI‐induced exosomal miR‐503 could disrupt mitochondrial homeostasis through the PGC‐1β/NRF‐1 and SIRT3/PDH/ATP synthase signalling pathways.

### 3.6 Endothelial cells are the primary source of circulating miR‐503 in EVs after AMI

To develop strategies for blocking miR‐503 biogenesis, the first step is to identify the origins of circulating miR‐503 in EVs after AMI. We stained the isolated serum‐derived EVs from AMI patients with the cell‐specific markers CD45, CD144, and CD90.2, representing EVs derived from haematopoietic cells, endothelial cells, and fibroblasts, respectively. We found that the levels of these markers in AMI‐EVs were similar to those in the control group (Figure S16A). Meanwhile, we isolated cardiomyocytes, fibroblasts, endothelial cells, and haematopoietic cells in cardiac tissue as previously described^22^ and measured the expression of mature miR‐503 and its precursor, pre‐miR‐503, in the respective cell population (Figure S16B). Mature miR‐503 expression was increased in these cell populations of AMI mice, while the expression of pre‐miR‐503 was increased only in endothelial cells after AMI, suggesting that endothelial cells are the major source of miR‐503 (Figure S16C).

Considering that hypoxia of endothelial cells is a critical event in the early AMI pathological process, we cultured MAECs under hypoxia and measured miR‐503 expression at the indicated time. We observed an efficient increase in miR‐503 expression 0.5 hour after hypoxia induction and then rapidly increased. We isolated EVs from MAECs after different periods of hypoxia and observed higher exosomal miR‐503 expression after 0.5‐6 hours of hypoxia (Figure S16D). Moreover, miR‐503 levels were increased in endothelial cell EVs but decreased by the specific miRNA inhibitor (Figure S16E). Therefore, early hypoxic endothelial cells are an important source of circulating exosomal miR‐503 after AMI.

**FIGURE 6:**
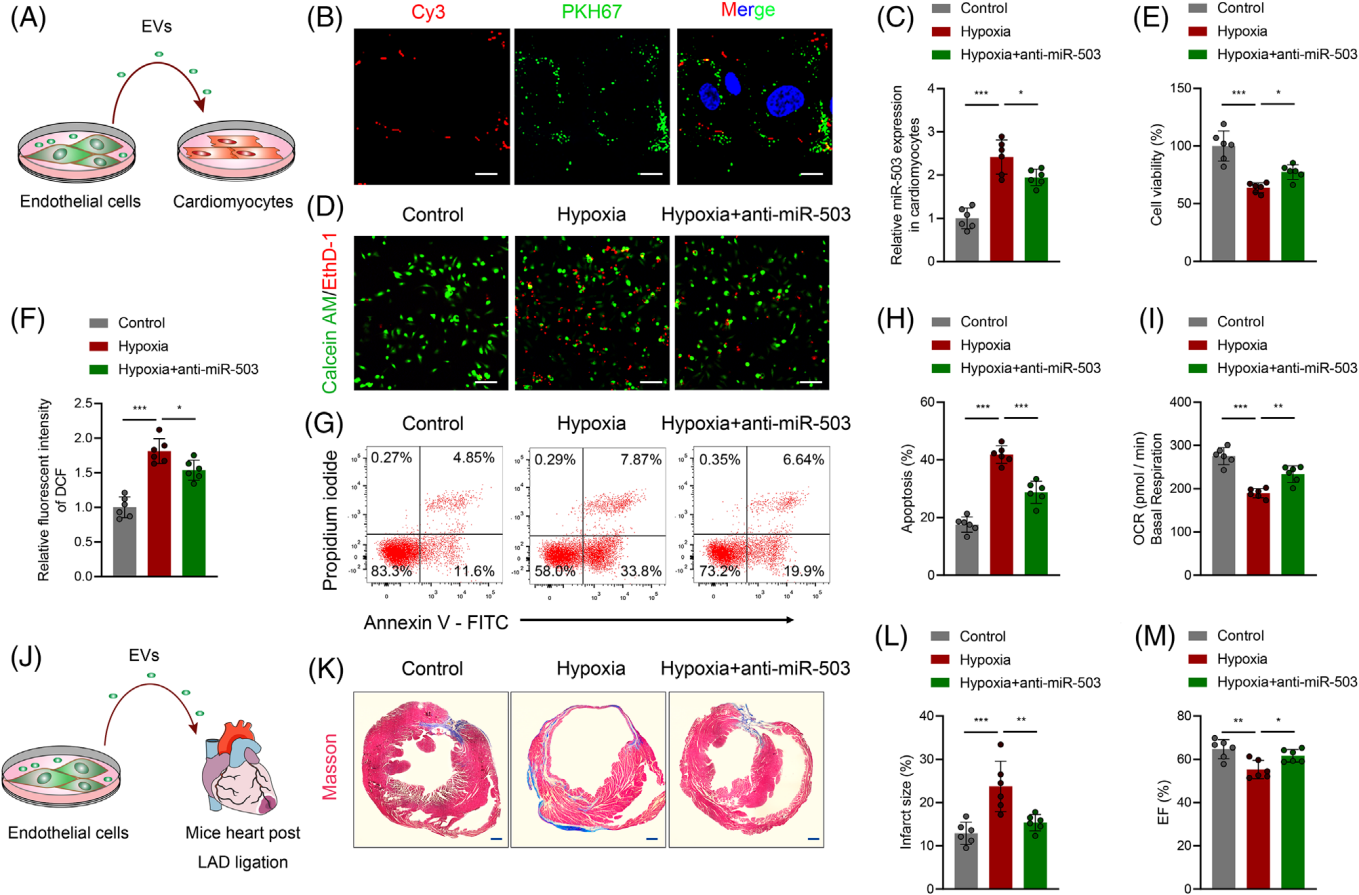
Identification of endothelial cells under hypoxia as an origin of exosomal miR‐503. (A) Scheme for extracellular vesicles (EVs) from endothelial cells in vitro. (B) Mouse arterial endothelial cells (MAECs) were transfected with Cy3‐labelled (red) miRNA negative control (NC) or miR‐503. Then, EVs were isolated from transfected MAECs and labelled with PKH67 dye (green) followed by incubation with cardiomyocytes. Cy3‐labelled miR‐503 was transported from MAECs to cardiomyocytes by EVs, as shown by confocal microscopy. Scale bar: 20 μm. (C) Real‐time quantitative polymerase chain reaction (qRT–PCR) analysis of miR‐503 expression in cardiomyocytes incubated with EVs from MAECs. (D, E) Calcein‐AM staining and ethidium homodimer‐1 staining of cardiomyocytes incubated with EVs from MAECs. Scale bar: 100 μm. Cellular viability was calculated and is shown in the right panel. (F) Reactive oxygen species (ROS) production was measured using a fluorescence microplate reader for the fluorescence intensity of DCF in the indicated groups. (G, H) Flow cytometry analysis of Annexin V‐APC and PI fluorescence in cardiomyocytes incubated with EVs from MAECs. The cellular apoptosis rate was calculated. (I) Oxygen consumption rate (OCR) levels in cardiomyocytes incubated with EVs from MAECs calculated by basal respiration. (J) Scheme for EVs from endothelial cells in vivo. (K, L) Representative images of cardiac sections with Masson's trichrome staining at 28 days after MI onset in mice treated with EVs from endothelial cells or pretreated with anti‐miR‐503 (left). Scale bar: 1 mm. The quantification of infarct size is shown (right). (M) Echocardiography assay analysis of cardiac function in mice after left anterior descending (LAD) ligation treated with EVs. (*n* = 6; *P < 0.05; **P < 0.01; ***P < 0.001)

We isolated EVs from endothelial cells, incubated them with cardiomyocytes, and then focused on the effects of exosomal miR‐503 derived from hypoxic endothelial cells on cardiomyocytes (Figure 6A). Confocal imaging identified the transport of exosomal Cy3‐labelled miR‐503 from MAECs to cardiomyocytes (Figure 6B). Subsequently, we found that cardiomyocytes treated with EVs under hypoxia exhibited increased miR‐503 expression, which decreased upon miR‐503 inhibitor pretreatment (Figure 6C). Notably, EVs derived from hypoxic endothelial cells induced cardiomyocyte viability loss, apoptosis, and mitochondrial metabolic disorders. However, the effect was improved by miR‐503 inhibition (Figure 6D‐I). Decreased PGC‐1β and SIRT3 expression levels and increased acetylation levels were observed in cardiomyocytes after treatment with EVs from hypoxic endothelial cells, whereas these effects were restored by miR‐503 inhibition (Figure S16F).

Next, we evaluated the role of miR‐503 in regulating MAEC functions. Successful miR‐503 transfection was confirmed by qRT–PCR (Figure S17A). Cell counting Kit‐8 assays and EdU staining indicated a significant decrease in cell growth after miR‐503 transfection (Figure S17B–E). Moreover, a higher apoptosis rate was observed in the miR‐503 expression‐treated cells (Figure S17F–G). Therefore, we concluded that miR‐503 could impair endothelial cell function by inhibiting cell proliferation and inducing apoptosis, which is consistent with the results of previous studies.^23^, ^24^

To directly identify whether hypoxic endothelial cell‐induced exosomal miR‐503 could exert adverse effects after AMI, we injected EVs isolated from MAECs under hypoxia or pretreated them with the inhibitor of miR‐503 (Figure 6J). A significant increase in the infarct size was observed after the injection of EVs under hypoxic conditions. Notably, miR‐503 inhibition diminished this effect (Figure 6K,L). An echocardiography assay suggested that cardiac function was aggravated after EVs were isolated from MAECs under hypoxia, but it was attenuated by miR‐503 inhibition (Figure 6M).

Therefore, endothelial cells are the primary source of circulating miR‐503, which impairs cardiomyocyte survival and accelerates pathology progression post MI. EVs serve as mediators of early hypoxia‐induced damage signalling from endothelial cells to cardiomyocytes after AMI.

### 3.7 Excessive miR‐503 maturation is mediated by METTL3 via N^6^‐m ethyladenosine

**FIGURE 7:**
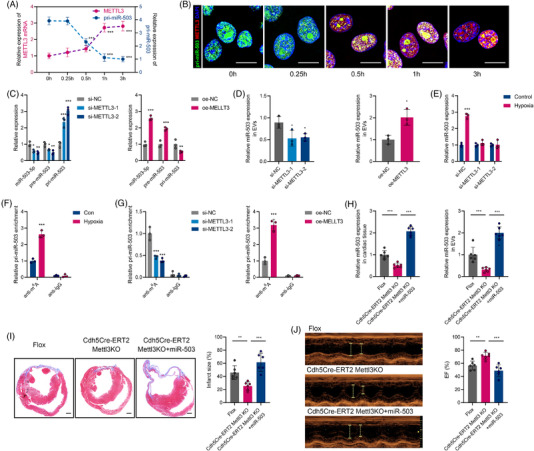
METTL3 could mediate pri‐miR‐503 maturation by N6‐methyladenosine under hypoxia. (A) Real‐time quantitative polymerase chain reaction (qRT–PCR) analysis of pri‐miR‐503 and METTL3 mRNA expression in mouse arterial endothelial cells (MAECs) under hypoxia at the indicated time points. (B) Colocalisation analysis between pri‐miR‐503 and METTL3 was detected by RNA fluorescence in situ hybridization (FISH) and immunofluorescence staining for MAECs under normoxia or hypoxia. Nuclei were stained with DAPI. Scale bar: 20 μm. (C) qRT–PCR analysis of pri‐miR‐503, pre‐miR‐503, and miR‐503 expression in MAECs transfected with si‐METTL3 or METTL3 overexpression plasmid. (D) Endothelial exosomal miR‐503 expression after si‐METTL3 or oe‐METTL3 transfection. (E) qRT–PCR analysis of pri‐miR‐503, pre‐miR‐503, and miR‐503 expression in MAECs transfected with si‐METTL3 under normoxia or hypoxia. (F) Analysis of m6A modification on pri‐miR‐503 by immunoprecipitation with an anti‐m^6^A antibody and qRT–PCR in MAECs under normoxia or hypoxia. (G) Analysis of m^6^A modification on pri‐miR‐503 by immunoprecipitation with an anti‐m^6^A antibody and qRT–PCR in MAECs transfected with si‐METTL3 or oe‐METTL3. (H) The expression of miR‐503 in EVs and cardiac tissue was analysed in endothelial *Mettl3* conditional knockout (cKO) mice, cKO mice treated with miR‐503, and littermate controls after myocardial infarction (MI) onset. (I) Representative images of cardiac sections with Masson's trichrome staining at 28 days after MI onset in the respective groups (left). Scale bar: 1 mm. The quantification of infarct size is shown (right). (J) Echocardiography assay analysis of cardiac function after MI onset in the respective groups (left). The percentage of left ventricular ejection fraction (EF) is shown (right). (*n* = 3 for in vitro studies and *n* = 6 for in vivo studies; *P < 0.05; **P < 0.01; ***P < 0.001)

Since endothelial cells are the “first responder” to hypoxia,^25^ while the pre‐miR‐503 was upregulated in endothelial cells under hypoxic conditions, we hypothesised that hypoxia may induce miR‐503 maturation. To elucidate the underlying mechanism, we analysed pri‐miR‐503 levels in MAECs exposed to hypoxia and observed prominently decreased pri‐miR‐503 levels compared to those in normoxic cells (Figure 7A). In addition, AMI‐derived endothelial cells showed decreased pri‐miR‐503 levels (Figure S18A), indicating that miR‐503 induction by hypoxia may be induced by aberrant pri‐miR‐503 processing and maturation after AMI.

METTL3, the first methyltransferase implicated in placing m^6^A on RNA, can modify pri‐miRNAs to produce mature miRNAs that affect cellular processes.^26^ Using the UCSC Genome Bioinformatics Site (http://genome.ucsc.edu/), we observed the potential m^6^A motif “RGAC” localised near the pri‐miR‐503 splicing site (Figure S18B). Using the m^6^A RNA immunoprecipitation assay (MeRIP) coupled with qRT–PCR analysis, we examined the level of potential sites binding with m^6^A. Using specific primers targeting the potential sites, we found that site three exhibited the best binding ability (Figure S18C). Therefore, we focused on site three in the following study.

We then examined whether METTL3‐mediated m^6^A modification affects hypoxia‐induced miR‐503 maturation. Remarkably, we detected a coincidence of hypoxia‐induced rapid METTL3 overexpression starting from 0.5 hour (Figure 7A) but not METTL14 or WTAP (Figure S18D). Meanwhile, METTLT3 levels were upregulated in cardiac endothelial cells after AMI (Figure S18E). Simultaneously, using double confocal imaging, we observed that METTL3 and pri‐miR‐503 were colocalized in endothelial cells under both normoxia and hypoxia. Moreover, we found that hypoxia induced a marked increase in METTL3 expression but decreased pri‐miR‐503 expression in the nucleus (Figure 7B).

We transfected METTL3 small interfering RNAs (si‐METTL3) or METTL3 overexpression plasmids (oe‐METTL3) into MAECs, and the METTL3 and m^6^A levels in the RNA extracted from METTL3 siRNAs were significantly decreased in the si‐METTL3 group compared to the control group but increased after METTL3 overexpression plasmid transfection (Figure S18F). Knockdown of *METTL3* in MAECs significantly reduced both the miR‐503 and pre‐miR‐503 levels but increased the pri‐miR‐503 level, while overexpression of *METTL3* markedly decreased the pri‐miR‐503 level but increased both miR‐503 and pre‐miR‐503 levels (Figure 7C). In addition, the exosomal miR‐503 levels in endothelial cells were decreased by si‐METTL3 and induced by oe‐METTL3 (Figure 7D). Moreover, METTL3 knockdown abolished hypoxia‐induced miR‐503 upregulation in endothelial cells (Figure 7E).

Subsequent MeRIP‐coupled qRT–PCR analysis revealed that pri‐miR‐503 m^6^A levels in hypoxic MAECs were significantly higher than those in normoxic control cells (Figure 7F). Moreover, MeRIP‐qRT–PCR revealed that METTL3 siRNAs significantly decreased the amount of the pri‐miR‐503 m^6^A motif. The METTL3 overexpression plasmid showed the opposite effect (Figure 7G). DGCR8 could further recruit the type III RNase DROSHA and yield pre‐miRNA products. We further identified that METTL3 depletion substantially decreased the interaction of DGCR8 with pri‐miR‐503. In contrast, the METTL3 overexpression plasmid increased this binding (Figure S18G). We constructed pri‐miR‐503 overexpression vectors containing m^6^A motif mutants and wild‐type. When the potential m^6^A motif in pri‐miR‐503 was mutated, the processing rate of pri‐miR‐503 to its mature form was prominently reduced (Figure S18H). Therefore, these findings suggest that hypoxia‐induced METTL3 upregulation could enhance the processing of endothelial exosomal miR‐503 biogenesis in an m^6^A‐dependent manner.

Moreover, mice that harboured floxed *Mettl3* and *Cdh5*‐promoter driven *CreERT2* were treated with tamoxifen to induce endothelial‐restricted deletion of *Mettl3*, as previously described.^27^, ^28^ We found that conditional endothelial *Mettl3* deletion decreased the expression of exosomal and cardiac miR‐503 following LAD ligation (Figure 7H). Using echocardiography assays and Masson's trichrome staining, we observed that conditional endothelial *Mettl3* deletion improved cardiac function and infarct size, whereas the injection of miR‐503 abolished these effects (Figure 7I,J). Overall, our data revealed a mechanism by which endothelial METTL3 regulates exosomal miR‐503 biogenesis and further triggers cardiac injury post AMI.

The deposition of m^6^A also requires a reader protein complex to recognise and process modified pri‐miRNAs. HNRNPA2B1 was confirmed to be a nuclear “reader” of the m^6^A mark and mature miRNA processing.^29^ Notably, transfection with shRNA targeting HNRNPA2B1 abolished hypoxia‐induced miR‐503 expression (Figure S18I). RIP‐coupled qRT–PCR showed that HNRNPA2B1 is bound to pri‐miR‐503 (Figure S18J). We then constructed an shRNA targeting HNRNPA2B1 (sh‐HNRNPA2B1) in MAECs (Figure S18K,L). Similar to METTL3 depletion, we found that transfection with sh‐HNRNPA2B1 effectively promoted pri‐miR‐503 levels but decreased the pre‐miR‐503 and miR‐503 levels (Figure S18M). In addition, exosomal miR‐503 levels were decreased by silencing HNRNPA2B1 (Figure S18N). RIP‐coupled qRT–PCR assays indicated that METTL3 depletion substantially decreased the interaction between endogenous HNRNPA2B1 and pri‐miR‐503. However, METTL3 overexpression promoted the binding of HNRNPA2B1 to pri‐miR‐503 (Figure S18O). Taken together, we confirmed that the METTL3‐mediated m^6^A mark is a key post‐transcriptional modification that promotes endothelial exosomal miR‐503 biogenesis via HNRNPA2B1 binding.

### 3.8 Early hypoxia‐induced H3K4me3 is responsible for activating METTL3 transcription in endothelial cells

**FIGURE 8:**
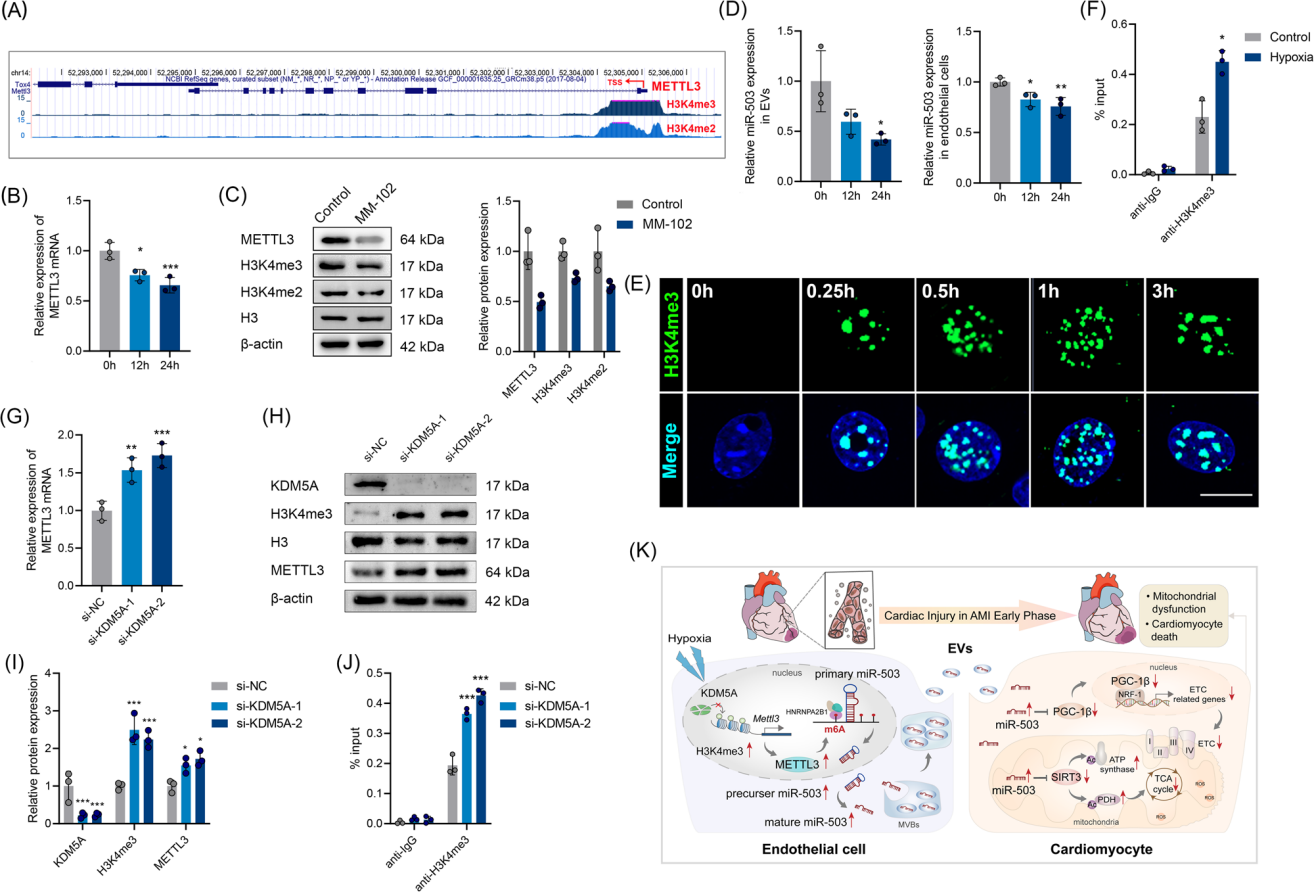
Hypoxia‐induced METTL3 promoter H3K4 methylation is the stem for endothelial extracellular vesicle (EV) dysfunction. (A) Data obtained from the UCSC genome bioinformatics site (http://genome.ucsc.edu/) exhibited high enrichment of H3K4me2 and H3K4me3 signals at the promoter of Mettl3. (B) Real‐time quantitative polymerase chain reaction (qRT–PCR) analysis of MELLT3 levels in mouse arterial endothelial cells (MAECs) treated with histone methyltransferase inhibitor (MM‐102) for 12 and 24 hours. (C) Western blot analysis of METTL3, H3K4me2, and H3K4me3 expression in MAECs treated with MM‐102 for 24 hours. (D) qRT–PCR analysis of exosomal or cellular miR‐503 expression in MAECs treated with MM‐102 for 12 and 24 hours. (E) Immunofluorescence staining for H3K4me3 in MAECs under hypoxia at the indicated times. Scale bar: 20 μm. (F) Chromatin immunoprecipitation (ChIP) assays were applied to detect H3K4me3 enrichment at the promoter of METTL3 in MAECs under normoxia or hypoxia. (G) qRT–PCR analysis of MELLT3 levels in MAECs transfected with si‐KDM5A. (H, I) Western blot analysis of KDM5A, H3K4me3, and METTL3 expression in MAECs transfected with si‐KDM5A. (J) ChIP assays were applied to detect H3K4me3 enrichment at the promoter of METTL3 in MAECs transfected with si‐KDM5A. (L) A proposed action model for this research (*n* = 3; *P < 0.05; **P < 0.01; ***P < 0.001)

To explore the mechanism underlying increased METTL3 expression in endothelial cells in response to hypoxia, we first analysed the modification of the *METTL3* promoter by UCSC. We found abundant H3K4 methylation in the promoter region of *METTL3* (Figure 8A). Then, we treated endothelial cells with an inhibitor of histone methyltransferase (HMTase inhibitor, MM‐102). We found that the *METTL3* mRNA and protein levels were significantly decreased in a time‐dependent manner (Figure 8B,C). The H3K4me3 and H3K4me2 levels were also decreased after treatment with MM‐102. Moreover, the miR‐503 levels in endothelial cells and EVs were reduced after treatment (Figure 8D). These results indicate that the histone methylase status is associated with METTL3 and miR‐503 levels.

Notably, oxygen can directly reprogram the chromatin and affect the expression of histone methylation‐related genes.^30^ Therefore, we analysed the histone methylation changes in response to short periods of hypoxia. We concluded that hypoxia induced a rapid increase in H3K4me3 but not H3K4me2 in MAECs (Figure S19A; Figure 8E). In addition, there were no obvious changes in the H3K4me3 levels in cardiomyocytes under hypoxia (Figure S19B). The chromatin immunoprecipitation (ChIP) assay results showed that the promoter region of *METTL3* was enriched in H3K4me3 signals under hypoxia (Figure 8F), indicating that hypoxia‐induced H3K4me3 activation in the promoter of *METTL3* may partly account for the upregulation of METTL3.

KDM5A, a histone demethylase, is oxygen‐sensitive. Hypoxia induces rapid chromatin reprogramming by suppressing KDM5A activation. To explore the role of KDM5A in modulating *METTL3* transcription, we knocked down KDM5A in MAECs and observed increased H3K4me3 and METTL3 levels (Figure 8G–I). Furthermore, KDM5A silencing increased H3K4me3 signals in the *METTL3* promoter (Figure 8J). All these data indicated that endothelial H3K4me3 could activate *METTL3* transcription and therefore promote m^6^A depending upon miR‐503 processing and exosomal miR‐503 production in the early phase of AMI.

## 4 DISCUSSION

In this study, we have made several novel discoveries. First, we revealed that EVs serve as early master regulators to exacerbate myocardial injury in AMI. Second, we illustrated that exosomal miR‐503 is a critical molecule that triggers mitochondrial dysfunction and cardiomyocyte death via the PGC‐1β/NRF‐1 and SIRT3/PDH/ATP synthase communication axes. Third, we clarified that the initiation and biogenesis of pathological exosomal miR‐503 are dependent on H3K4me3/METTL3‐mediated m^6^A modification of pri‐miR‐503 in cardiac endothelial cells during the early phase of AMI.

The pathological course of AMI is associated with a wide range of intercellular communications, thus incurring cardiac dysfunction, acute cardiac arrest, and even sudden death. Due to the sizable knowledge gap between therapeutic strategies and irreversible cardiomyocyte loss in complex ischemic myocardial scenarios, AMI has a substantial impact on global health. Recently, the critical role of EV transfer among various cardiac cellular components has been emphasised in AMI physiopathology.^5^ It has been proposed that EVs accumulated in the ischemic myocardium regulate cardiac inflammation by stimulating monocyte cytokine secretion after AMI.^31^ The endothelium‐derived EVs and myocardial EVs mobilise splenic monocytes and bone marrow progenitor cells in MI, respectively.^32^, ^33^ Moreover, the infarcted myocardial‐derived EVs could promote angiogenesis.^34^ These studies indicate that EV shuttling in circulation is an effective communicator of biological signals in the pathological process of AMI. However, the effect of endogenous EVs on cardiomyocyte injury after AMI remains unknown. In the present study, we reported a previously undefined regulatory mechanism by which acute injury “signals” cardiomyocytes during myocardial ischemia. We provided the first evidence that EVs are critical mediators that exacerbate ischemic cardiac injury in AMI. Strategies for preventing or blocking adverse EV production may have strong potential protective effects against AMI‐related cardiomyocyte loss.

EV shuttling is an effective mode of transport for biomolecules, including miRNAs, mRNAs, and proteins, through systemic circulation to regulate cell biology, including cardiomyocyte apoptosis and survival.^35^, ^36^ Therefore, the circulating EV numbers and content constitute novel markers for cardiovascular risk and myocardial injury.^37^ MI–reperfusion‐induced exosomal miR‐155 is critical for aggravating cardiac injury.^38^ Moreover, the pathological condition of heart failure reprogrammed the exosomal cargos and induced miR‐21‐5p expression, which impaired the regenerative potential.^36^ However, AMI‐triggered changes in the content of EVs are unclear. Using deep sequencing, we mapped gene expression profiles in the patients with AMI and the control group. Among the differentially expressed genes, miR‐503 attracted our attention. In previous cardiovascular studies, miR‐503 was reported to be a critical regulator of early cardiac fate and could modulate cardiomyocyte apoptosis via the PI3K/Akt pathway.^39^, ^40^ However, the role of miR‐503 in AMI progression requires further exploration. The function of miR‐503 encapsulated in EVs and shutting in circulation is yet to be deciphered. In this study, the expression of exosomal miR‐503 was significantly increased in the AMI group and was associated with the degree of myocardial injury. Further functional studies confirmed that exosomal miR‐503 is an early specific molecule that mediates cardiac damage. These observations are compelling, as circulating exosomal miR‐503 can not only indicate early myocardial injury but also serve as a functional molecule to accelerate cardiomyocyte loss.

Mitochondria, the energy‐producing engines, are the principal organelles responsible for energy export and metabolism homeostasis of the heart.^41^ More importantly, mitochondrial status is closely associated with cellular survival, and mitochondrial metabolic dysfunction is an early event during cardiac injury in AMI.^4^ PGC‐1s can broadly modulate the transcriptional regulation of mitochondrial biogenesis and have thus been identified as powerful regulators to maintain mitochondrial functions in cardiomyocytes.^18^, ^42^ We demonstrated that PGC‐1β levels were downregulated in the cardiac tissue of mice after AMI, especially in those injected with EVs derived from AMI. Furthermore, we identified *PGC‐1β* as a direct target gene of miR‐503. Mechanistically, we suggested that AMI‐derived EVs contained abundant miR‐503, which might directly control the PGC‐1β/NRF‐1 axis to disrupt the expression of ETC complex‐related genes, thereby triggering mitochondrial dysfunction.

Moreover, the maintenance of mitochondrial function and cell viability depends on the state of mitochondrial lysine acetylation modification.^43^ Sirtuin 3 (SIRT3), a typical sirtuin, is a prominent deacetylase that modulates acetylation levels in mitochondria by targeting a series of metabolic enzymes.^17^ SIRT3 is reportedly downregulated after heart failure. Loss of SIRT3 impairs cardiac energy metabolism and induces apoptosis mainly by promoting ROS production and suppressing OXPHOS.^20^ We also suggest that SIRT3 is a direct target gene of miR‐503 and that abundant AMI‐derived exosomal miR‐503 directly suppresses SIRT3 in cardiomyocytes and further acetylates PDH and ATP synthase to compromise their activity as well as impair mitochondrial function. Therefore, SIRT3 suppression and downstream acetylation modification are possible molecular regulatory mechanisms by which exosomal miR‐503 accelerates cardiomyocyte apoptosis and death.

Although previous studies have proposed the function of exosomal miRNAs in intercellular and intertissue crosstalk, the contribution of various cell types to the circulating exosomal miRNA pool remains largely unclear. To investigate the origin and mechanism of the dramatic increase in circulating exosomal miR‐503 in the early phase of AMI, we sorted different cell types in the cardiac tissue and identified endothelial cell type as a potential source of exosomal miR‐503. Endothelial cell hypoxia could trigger quick miR‐503 biosynthesis as well as exosomal miR‐503 transport. This attracted our attention since vascular endothelial cells, regarded as the “first responder” to external stimuli, can not only adjust their status but also modulate cardiomyocyte and other noncardiomyocyte functions by secreting some potential active components.^25^, ^44^, ^45^ It has been reported that knockout of the prolyl hydroxylase domain, the key molecule in the hypoxia‐inducible factor pathway, promotes cardiomyocyte proliferation and prevents heart failure after MI.^25^ Since EVs can reflect the pathophysiological state of producing cells, EVs may confer adverse injury factors to cardiomyocytes under hypoxia. Interestingly, a recent study highlighted that normoxic and hypoxic endothelial cell EVs had similar protein landscapes,^46^ suggesting other biomolecular cargo responses for this effect. Therefore, we hypothesised that hypoxia induced exosomal miR‐503 transfers from endothelial cells to cardiomyocytes to accelerate cardiomyocyte injury. To confirm this, we isolated EVs from endothelial cells with hypoxic stimulation and incubated them with cardiomyocytes and found that these EVs showed impaired cardiomyocyte viability and induced cell apoptosis, whereas this effect was reversed by miR‐503 pretreatment. Therefore, it is reasonable to conclude that EVs are important messengers mediating the pathological communication between vascular endothelial cells and cardiomyocytes to promote myocardial injury via miR‐503 transfer. The horizontal spreading of these early “hypoxia signals” brings cardiomyocytes into the hypoxia‐like scenario, therefore exerting an impaired role.

The initial event for miRNA biogenesis is the recognition of the primary miRNA hairpin by DGCR8.^47^ It has been demonstrated that m^6^A enriched in pri‐miRNA transcripts could facilitate this recognition and processing, thus promoting miRNA biogenesis.^26^ The deposit of m^6^A requires “writers,” including METTL3, “erasers,” including WTAP, and “readers” including YTHDF1 to produce mature miRNAs that exert functional effects. METTL3, the first identified m^6^A reader, is the main methyltransferase‐inducing methylation process.^48^, ^49^ Although the role of METTL3 in modulating cancer cell survival and apoptosis has been well described, the role of METTL3 in MI is yet to be explored. In the present study, we confirmed that in the early AMI phase, METTL3 accumulation was induced and could methylate pri‐miR‐503, which further triggered the maturation of pri‐miR‐503 and exosomal miR‐503 production in endothelial cells. Furthermore, we suggest that the binding of HNRNPA2B1 to pri‐miR‐503 m^6^A marks could further recruit the microprocessor complex and result in pri‐miR‐503 processing. Therefore, m^6^A epigenetic modification is an early phase regulatory mechanism that controls exosomal miR‐503 biosynthesis in AMI. Since m^6^A modification exists in a series of primary miRNAs biogenesis, it would be valuable to clarify whether the m^6^A mark could promote other miRNAs during MI progression and therefore play a wide role for MI‐specific miRNAs processing as a “signal amplifier.”

Endothelial cells, which represent the largest noncardiomyocyte cell subset within cardiac tissue, are sensitive to hypoxia.^50^ To adapt to lower oxygen levels, a specific cellular transcriptional programme is activated.^51^ The oxygen level controls chromatin regulators to determine cell fate.^30^ Recent studies have reported that histone methylation is rapidly induced in response to hypoxia, which is hypoxia inducible–factor independent.^52^ In this study, we found that early hypoxia‐induced H3K4me3 is responsible for activating *METTL3* transcription, leading to exosomal miR‐503 production in endothelial cells after AMI. Hypoxia is an aetiological factor in chromatin reprogramming. Histone methylation modification is linked to oxygen sensitivity and cell fate. It would be compelling to identify whether histone methylation changes play a role in other endothelial cell hypoxia‐related coronary artery diseases. An emerging pathological significance of EVs is to ferry “prototype” epigenetic alterations from donor cells to recipient cells deficient in these messages.^53^, ^54^ Strategies preventing exosomal miR‐503 biogenesis from the initial epigenetic modification level may provide strategies for the treatment of early myocardial injury after AMI.

There are several limitations to this study that should be acknowledged. We identified the reason for the circulating exosomal miR‐503 overexpression. The underlying mechanism of miR‐503 sorting into EVs remains unclear. Regarding exosomal molecular loading, HNRNPA2B1 was first identified to regulate the export of miRNAs.^55^, ^56^ However, miR‐503 did not have a specific recognition motif. Furthermore, changes in miRNA target genes could also influence miRNA sorting into EVs. Therefore, the specific sequences of miRNAs may guide their incorporation into EVs. Second, although we showed that miR‐503‐targeted therapy in AMI patients seems highly feasible and the measurement of blood test for circulating miR‐503 in EVs would enable the accurate selection of individuals who may benefit from this therapy, we did not determine whether other miRNAs may participate in this process. Third, we identified endothelial cells as the origin of exosomal miR‐503 by analysing cardiac cell types. Since AMI can activate the systemic response, other nonmyocardial cells may exist, which requires further study. Moreover, endothelial cell‐specific miR‐503 knockdown mice need to be studied further. Finally, in this study, we focused on the direct interaction of circulating EVs with cardiomyocytes. These EVs may also be taken up by other cell types, such as cardiac immune cells, and in return regulate cardiomyocytes by cytokines or even EVs. In the past decade, cellular communication in MI and strategies for optimising myocardial protection have been extensively studied. A complete understanding of the system crosstalk would provide more therapeutic strategies against ischemic heart disease.

## 5 CONCLUSIONS

We identified an endogenous myocardial injury mechanism mediated by EVs in the early phase of AMI, in which the vicious axes of PGC‐1β/NRF‐1 and SIRT3/PDH/ATP synthase are involved. Vascular endothelial cells, a priority for sensing hypoxia, may be a predominant source of exosomal miR‐503, which activates the H3K4me3/METTL3‐mediated m^6^A mark. Cutting off miR‐503 biogenesis from the origin or using miR‐503 antagonists may be new effective therapeutic interventions blocking EV‐mediated pathological communications between endothelial cells and cardiomyocytes, providing early cardioprotection after AMI.

## CONFLICT OF INTEREST

The authors declare no competing interests.

## Supporting information

Supporting Information

## References

[1] GBDCoD Collaborators . Global, regional, and national age‐sex‐specific mortality for 282 causes of death in 195 countries and territories, 1980–2017: a systematic analysis for the global burden of disease study 2017. Lancet. 2018;392(10159):1736‐1788. 30496103 10.1016/S0140-6736(18)32203-7 PMC6227606

[2] Reed GW , Rossi JE , Cannon CP . Acute myocardial infarction. Lancet. 2017;389(10065):197‐210. 27502078 10.1016/S0140-6736(16)30677-8

[3] Marenzi G , Cosentino N , Boeddinghaus J , et al. Diagnostic and prognostic utility of circulating cytochrome c in acute myocardial infarction. Circ Res. 2016;119(12):1339‐1346. 27799252 10.1161/CIRCRESAHA.116.309792 PMC5627527

[4] Ramachandra CJA , Hernandez‐Resendiz S , Crespo‐Avilan GE , Lin YH , Hausenloy DJ . Mitochondria in acute myocardial infarction and cardioprotection. EBioMedicine. 2020;57:102884. 32653860 10.1016/j.ebiom.2020.102884 PMC7355051

[5] Martins‐Marques T , Hausenloy DJ , Sluijter JPG , Leybaert L , Girao H . Intercellular communication in the heart: therapeutic opportunities for cardiac ischemia. Trends Mol Med. 2021;27(3):248‐262. 33139169 10.1016/j.molmed.2020.10.002

[6] Barile L , Moccetti T , Marban E , Vassalli G . Roles of exosomes in cardioprotection. Eur Heart J. 2017;38(18):1372‐1379. 27443883 10.1093/eurheartj/ehw304

[7] Sahoo S , Adamiak M , Mathiyalagan P , Kenneweg F , Kafert‐Kasting S , Thum T . Therapeutic and diagnostic translation of extracellular vesicles in cardiovascular diseases: roadmap to the clinic. Circulation. 2021;143(14):1426‐1449. 33819075 10.1161/CIRCULATIONAHA.120.049254 PMC8021236

[8] Makarova J , Turchinovich A , Shkurnikov M , Tonevitsky A . Extracellular miRNAs and cell‐cell communication: problems and prospects. Trends Biochem Sci. 2021;46(8):640‐651. 33610425 10.1016/j.tibs.2021.01.007

[9] Mori MA , Ludwig RG , Garcia‐Martin R , Brandao BB , Kahn CR . Extracellular miRNAs: from biomarkers to mediators of physiology and disease. Cell Metab. 2019;30(4):656‐673. 31447320 10.1016/j.cmet.2019.07.011 PMC6774861

[10] Collet JP , Thiele H , Barbato E , et al. 2020 ESC guidelines for the management of acute coronary syndromes in patients presenting without persistent ST‐segment elevation. Eur Heart J. 2021;42(14):1289‐1367. 32860058 10.1093/eurheartj/ehaa575

[11] Task Force M , Montalescot G , Sechtem U , et al. 2013 ESC guidelines on the management of stable coronary artery disease: the Task Force on the management of stable coronary artery disease of the European Society of Cardiology. Eur Heart J. 2013;34(38):2949‐3003. 23996286 10.1093/eurheartj/eht296

[12] Dutta P , Courties G , Wei Y , et al. Myocardial infarction accelerates atherosclerosis. Nature. 2012;487(7407):325‐329. 22763456 10.1038/nature11260 PMC3401326

[13] Au Yeung CL , Co NN , Tsuruga T , et al. Exosomal transfer of stroma‐derived miR21 confers paclitaxel resistance in ovarian cancer cells through targeting APAF1. Nat Commun. 2016;7:11150. 27021436 10.1038/ncomms11150 PMC4820618

[14] Thomou T , Mori MA , Dreyfuss JM , et al. Adipose‐derived circulating miRNAs regulate gene expression in other tissues. Nature. 2017;542(7642):450‐455. 28199304 10.1038/nature21365 PMC5330251

[15] Coumans FAW , Brisson AR , Buzas EI , et al. Methodological guidelines to study extracellular vesicles. Circ Res. 2017;120(10):1632‐1648. 28495994 10.1161/CIRCRESAHA.117.309417

[16] Thery C , Witwer KW , Aikawa E , et al. Minimal information for studies of extracellular vesicles 2018 (MISEV2018): a position statement of the International Society for Extracellular Vesicles and update of the MISEV2014 guidelines. J Extracell Vesicles. 2018;7(1):1535750. 30637094 10.1080/20013078.2018.1535750 PMC6322352

[17] Zhang J , Xiang H , Liu J , Chen Y , He RR , Liu B . Mitochondrial sirtuin 3: new emerging biological function and therapeutic target. Theranostics. 2020;10(18):8315‐8342. 32724473 10.7150/thno.45922 PMC7381741

[18] Rowe GC , Jiang A , Arany Z . PGC‐1 coactivators in cardiac development and disease. Circ Res. 2010;107(7):825‐838. 20884884 10.1161/CIRCRESAHA.110.223818 PMC2955978

[19] Wu Z , Puigserver P , Andersson U , et al. Mechanisms controlling mitochondrial biogenesis and respiration through the thermogenic coactivator PGC‐1. Cell. 1999;98(1):115‐124. 10412986 10.1016/S0092-8674(00)80611-X

[20] Zhang X , Ji R , Liao X , et al. MicroRNA‐195 regulates metabolism in failing myocardium via alterations in sirtuin 3 expression and mitochondrial protein acetylation. Circulation. 2018;137(19):2052‐2067. 29330215 10.1161/CIRCULATIONAHA.117.030486 PMC6449058

[21] Jung JH , Ikeda G , Tada Y , et al. miR‐106a‐363 cluster in extracellular vesicles promotes endogenous myocardial repair via Notch3 pathway in ischemic heart injury. Basic Res Cardiol. 2021;116(1):19. 33742276 10.1007/s00395-021-00858-8 PMC8601755

[22] Rogg EM , Abplanalp WT , Bischof C , et al. Analysis of cell type‐specific effects of microrna‐92a provides novel insights into target regulation and mechanism of action. Circulation. 2018;138(22):2545‐2558. 30571345 10.1161/CIRCULATIONAHA.118.034598

[23] Kim J , Kang Y , Kojima Y , et al. An endothelial apelin‐FGF link mediated by miR‐424 and miR‐503 is disrupted in pulmonary arterial hypertension. Nat Med. 2013;19(1):74‐82. 23263626 10.1038/nm.3040 PMC3540168

[24] Caporali A , Meloni M , Vollenkle C , et al. Deregulation of microRNA‐503 contributes to diabetes mellitus‐induced impairment of endothelial function and reparative angiogenesis after limb ischemia. Circulation. 2011;123(3):282‐291. 21220732 10.1161/CIRCULATIONAHA.110.952325

[25] Fan Q , Mao H , Angelini A , et al. Depletion of endothelial prolyl hydroxylase domain protein 2 and 3 promotes cardiomyocyte proliferation and prevents ventricular failure induced by myocardial infarction. Circulation. 2019;140(5):440‐442. 31356139 10.1161/CIRCULATIONAHA.118.039276 PMC6699770

[26] Alarcon CR , Lee H , Goodarzi H , Halberg N , Tavazoie SF . N6‐methyladenosine marks primary microRNAs for processing. Nature. 2015;519(7544):482‐485. 25799998 10.1038/nature14281 PMC4475635

[27] Li X , Yuan B , Lu M , et al. The methyltransferase METTL3 negatively regulates nonalcoholic steatohepatitis (NASH) progression. Nat Commun. 2021;12(1):7213. 34893641 10.1038/s41467-021-27539-3 PMC8664922

[28] Hao H , Hu S , Chen H , et al. Loss of endothelial CXCR7 impairs vascular homeostasis and cardiac remodelling after myocardial infarction: implications for cardiovascular drug discovery. Circulation. 2017;135(13):1253‐1264. 28154007 10.1161/CIRCULATIONAHA.116.023027

[29] Alarcon CR , Goodarzi H , Lee H , Liu X , Tavazoie S , Tavazoie SF . HNRNPA2B1 is a mediator of m(6)A‐dependent nuclear RNA processing events. Cell. 2015;162(6):1299‐1308. 26321680 10.1016/j.cell.2015.08.011 PMC4673968

[30] Batie M , Frost J , Frost M , Wilson JW , Schofield P , Rocha S . Hypoxia induces rapid changes to histone methylation and reprograms chromatin. Science. 2019;363(6432):1222‐1226. 30872526 10.1126/science.aau5870

[31] Loyer X , Zlatanova I , Devue C , et al. Intra‐cardiac release of extracellular vesicles shapes inflammation following myocardial infarction. Circ Res. 2018;123(1):100‐106. 29592957 10.1161/CIRCRESAHA.117.311326 PMC6023578

[32] Akbar N , Digby JE , Cahill TJ , et al. Endothelium‐derived extracellular vesicles promote splenic monocyte mobilization in myocardial infarction. JCI Insight. 2017;2(17). 10.1172/jci.insight.93344 PMC5621885 28878126

[33] Cheng M , Yang J , Zhao X , et al. Circulating myocardial microRNAs from infarcted hearts are carried in exosomes and mobilise bone marrow progenitor cells. Nat Commun. 2019;10(1):959. 30814518 10.1038/s41467-019-08895-7 PMC6393447

[34] Li H , Liao Y , Gao L , et al. Coronary serum exosomes derived from patients with myocardial ischemia regulate angiogenesis through the miR‐939‐mediated nitric oxide signaling pathway. Theranostics. 2018;8(8):2079‐2093. 29721064 10.7150/thno.21895 PMC5928872

[35] Garikipati VNS , Shoja‐Taheri F , Davis ME , Kishore R . Extracellular vesicles and the application of system biology and computational modeling in cardiac repair. Circ Res. 2018;123(2):188‐204. 29976687 10.1161/CIRCRESAHA.117.311215 PMC6512976

[36] Qiao L , Hu S , Liu S , et al. microRNA‐21‐5p dysregulation in exosomes derived from heart failure patients impairs regenerative potential. J Clin Invest. 2019;129(6):2237‐2250. 31033484 10.1172/JCI123135 PMC6546482

[37] Sluijter JPG , Davidson SM , Boulanger CM , et al. Extracellular vesicles in diagnostics and therapy of the ischaemic heart: position Paper from the Working Group on Cellular Biology of the Heart of the European Society of Cardiology. Cardiovasc Res. 2018;114(1):19‐34. 29106545 10.1093/cvr/cvx211 PMC5852624

[38] Ge X , Meng Q , Wei L , et al. Myocardial ischemia‐reperfusion induced cardiac extracellular vesicles harbour proinflammatory features and aggravate heart injury. J Extracell Vesicles. 2021;10(4):e12072. 33664937 10.1002/jev2.12072 PMC7902529

[39] Zhu W , Lei J , Bai X , Wang R , Ye Y , Bao J . MicroRNA‐503 regulates hypoxia‐induced cardiomyocytes apoptosis through PI3K/Akt pathway by targeting IGF‐1R. Biochem Biophys Res Commun. 2018;506(4):1026‐1031. 30404731 10.1016/j.bbrc.2018.10.160

[40] Shen X , Soibam B , Benham A , et al. miR‐322/‐503 cluster is expressed in the earliest cardiac progenitor cells and drives cardiomyocyte specification. Proc Natl Acad Sci U S A. 2016;113(34):9551‐9556. 27512039 10.1073/pnas.1608256113 PMC5003281

[41] Bonora M , Wieckowski MR , Sinclair DA , Kroemer G , Pinton P , Galluzzi L . Targeting mitochondria for cardiovascular disorders: therapeutic potential and obstacles. Nat Rev Cardiol. 2019;16(1):33‐55. 30177752 10.1038/s41569-018-0074-0 PMC6349394

[42] Montaigne D , Butruille L , Staels B . PPAR control of metabolism and cardiovascular functions. Nat Rev Cardiol. 2021;18(12):809‐823. 34127848 10.1038/s41569-021-00569-6

[43] Winnik S , Auwerx J , Sinclair DA , Matter CM . Protective effects of sirtuins in cardiovascular diseases: from bench to bedside. Eur Heart J. 2015;36(48):3404‐3412. 26112889 10.1093/eurheartj/ehv290 PMC4685177

[44] Yue Y , Wang C , Benedict C , et al. Interleukin‐10 deficiency alters endothelial progenitor cell‐derived exosome reparative effect on myocardial repair via integrin‐linked kinase enrichment. Circ Res. 2020;126(3):315‐329. 31815595 10.1161/CIRCRESAHA.119.315829 PMC7015105

[45] Chen W , Spitzl A , Mathes D , et al. Endothelial actions of ANP enhance myocardial inflammatory infiltration in the early phase after acute infarction. Circ Res. 2016;119(2):237‐248. 27142162 10.1161/CIRCRESAHA.115.307196

[46] Yadid M , Lind JU , Ardona HAM , et al. Endothelial extracellular vesicles contain protective proteins and rescue ischemia‐reperfusion injury in a human heart‐on‐chip. Sci Transl Med. 2020;12(565):eaax8005. 10.1126/scitranslmed.aax8005 PMC8969368 33055246

[47] Ha M , Kim VN . Regulation of microRNA biogenesis. Nat Rev Mol Cell Biol. 2014;15(8):509‐524. 25027649 10.1038/nrm3838

[48] Meyer KD , Jaffrey SR . The dynamic epitranscriptome: n6‐methyladenosine and gene expression control. Nat Rev Mol Cell Biol. 2014;15(5):313‐326. 24713629 10.1038/nrm3785 PMC4393108

[49] Jiang X , Liu B , Nie Z , et al. The role of m6A modification in the biological functions and diseases. Signal Transduct Target Ther. 2021;6(1):74. 33611339 10.1038/s41392-020-00450-x PMC7897327

[50] Pinto AR , Ilinykh A , Ivey MJ , et al. Revisiting cardiac cellular composition. Circ Res. 2016;118(3):400‐409. 26635390 10.1161/CIRCRESAHA.115.307778 PMC4744092

[51] Li Z , Solomonidis EG , Meloni M , et al. Single‐cell transcriptome analyses reveal novel targets modulating cardiac neovascularization by resident endothelial cells following myocardial infarction. Eur Heart J. 2019;40(30):2507‐2520. 31162546 10.1093/eurheartj/ehz305 PMC6685329

[52] Chakraborty AA , Laukka T , Myllykoski M , et al. Histone demethylase KDM6A directly senses oxygen to control chromatin and cell fate. Science. 2019;363(6432):1217‐1222. 30872525 10.1126/science.aaw1026 PMC7336390

[53] Ramakrishnaiah V , Thumann C , Fofana I , et al. Exosome‐mediated transmission of hepatitis C virus between human hepatoma Huh7.5 cells. Proc Natl Acad Sci. 2013;110(32):13109‐13113. 23878230 10.1073/pnas.1221899110 PMC3740869

[54] Yan W , Wu X , Zhou W , et al. Cancer‐cell‐secreted exosomal miR‐105 promotes tumour growth through the MYC‐dependent metabolic reprogramming of stromal cells. Nat Cell Biol. 2018;20(5):597‐609. 29662176 10.1038/s41556-018-0083-6 PMC5920728

[55] Villarroya‐Beltri C , Baixauli F , Gutierrez‐Vazquez C , Sanchez‐Madrid F , Mittelbrunn M . Sorting it out: regulation of exosome loading. Semin Cancer Biol. 2014;28:3‐13. 24769058 10.1016/j.semcancer.2014.04.009 PMC4640178

[56] Villarroya‐Beltri C , Gutierrez‐Vazquez C , Sanchez‐Cabo F , et al. Sumoylated hnRNPA2B1 controls the sorting of miRNAs into exosomes through binding to specific motifs. Nat Commun. 2013;4:2980. 24356509 10.1038/ncomms3980 PMC3905700

