# Supplementary material for: Extracellular vesicle‐packaged mitochondrial disturbing miRNA exacerbates cardiac injury during acute myocardial infarction
Source: Clin Transl Med. 2022 Apr 22;12(4):e779. doi: 10.1002/ctm2.779 (PMC9028097; doi:10.1002/ctm2.779)
Supplement: Supplementary file 1 — Supporting Information [file CTM2-12-e779-s001.pdf]

Supplementary Materials for

Extracellular vesicles-packaged mitochondrial disturbing miRNA exacerbates cardiac injury during acute myocardial infarction

Ping Sun[1,2], Chao Wang[1,2], Ge Mang[2,3], Xiangli Xu[4], Shuai Fu[1,2], Jianfeng Chen[5], Xiaoqi Wang[2,3], Weiwei Wang[2,3], Hairu Li[1,2], Peng Zhao[1], Yifei Li[1,2], Qi Chen[2,3], Naixin Wang[2,3], Zhonghua Tong[2,3], Xin Fu[1], Ying Lang[1,2], Shasha Duan[1,2], Dongmei Liu[1], Maomao Zhang[2,3*], Jiawei Tian[1,2*]

1. Department of Ultrasound, the Second Affiliated Hospital of Harbin Medical University, Harbin, Heilongjiang Province, China.

2. The Key Laboratory of Myocardial Ischemia, Harbin Medical University, Ministry of Education, Harbin, Heilongjiang Province, China.

3. Department of Cardiology, the Second Affiliated Hospital of Harbin Medical University, Harbin, Heilongjiang Province, China.

4. Department of Ultrasound, the Second Hospital of Harbin city, Harbin, Heilongjiang Province, China.

5. Laboratory Animal Center, the Second Affiliated Hospital of Harbin Medical University, Harbin, Heilongjiang Province, China.

[*]Corresponding to:

Dr. Jiawei Tian. Email: jwtian2004@163.com

Dr. Maomao Zhang. Email: maomaolp1983@163.com

Supplementary information

Materials and Methods

Sequencing and bioinformatics

5ug of RNA was extracted from exosomes purified per sample. Both 3' and 5' adaptors were ligated to each end. Reverse transcription and polymerase chain reaction (PCR) amplifications were performed. The cDNA construct was purified by electrophoresis and sequenced on an Illumina HiSeq 2500 platform (OE Biotechnology Co., Ltd, Shanghai, China). The known miRNAs were identified by aligning against the miRBase v.21 database (http://www.mirbase.org/). Differentially expressed genes (DEGs) were identified under the threshold of p value < 0.05 and fold change >2. The *p*-value was calculated with the DEG algorithm using R package.

Cell culture and transfection

AC16 human cardiomyocyte-like cells were a kind gift from the College of Pharmacy at Harbin Medical University. Mouse aortic endothelial cells (MAEC) were purchased from the Chinese Academy of Science (Shanghai, China). Neonatal mouse cardiomyocytes (NMCMs) were isolated from 1-3 days old neonatal C57BL/6 mice as previously described [1]. AC16 cells, MAECs, and NMCMs were maintained in Dulbecco's modified Eagle's medium (DMEM; Gibco, USA) medium supplemented with 10% FBS (Gibco, USA). The cells were grown without antibiotics in an atmosphere of 5% CO_2 and 99% relative humidity at 37°C. Hypoxia experiments were conducted in a controlled atmosphere chamber (Lansing, MI, USA).

For cellular transfection studies, the miRNA mimics were designed as a double sequence according to the stranded sequence of the mature miRNA sequence and its complementary sequence. The anti-miRNAs were designed as single-stranded sequences of reverse complementary sequences of mature miRNA sequences and were modified by full-chain methylation (RiboBio Co., Ltd. Guangzhou, China). The sequences of synthetic miRNA mimics and inhibitor were listed in Table S3. The SIRT3 and METTL3 coding sequences were cloned into the pcDNA3.1. Empty vectors served as negative controls (GenePharma, Shanghai, China). siRNAs and shRNAs were designed and synthesized by RiboBio and GenePharma. These sequences are listed in Table S2. Transfection was performed using Lipofectamine 3000 (Invitrogen) and Opti-MEM serum-free medium (Gibco, Grand Island, NY, USA), according to the manufacturer's instructions. The cell medium was replaced with DMEM complete medium at 8h after transfection. The following assays were performed 48h after transfection.

Exosomal transfection of miRNA mimics or anti-miRNAs was performed using an Exo-Fect Exosome Transfection Kit (System Biosciences). Briefly, the transfected EV system consisted of 10μl of Exo-Fect solution, 20pmol of miRNAs or anti-miRNAs, 50μl of EVs, and 70μl of PBS. The transfection system was incubated at 37°C for 10 min, followed by mixing with 30μl the ExoQuick-TC at 4°C for 30 minutes. The EVs were collected by centrifugated at 13000 r.p.m for 3 minutes and resuspended in 300μl of PBS. Finally, 150μl of EVs after transfection was incubated with 1×10^5 cells.

RNA extraction, reverse transcription, and quantitative RT-PCR

Exosomal and cellular RNAs were extracted using the miRcute miRNA Isolation Kit (TIANGEN BIOTECH, China), following the manufacturer's protocol. Subsequently, 50ng of isolated RNA was reverse-transcribed into cDNA using an miRNA First-Strand cDNA Synthesis Kit (TIANGEN BIOTECH). Quantitative RT-PCR was performed using the miRCute Enhanced microRNA Fluorescence Quantitative Assay Kit (SYBR Green) (TIANGEN BIOTECH). 5 fmol/μl synthetic cel-miR-39 served as an external control for exosomal miRNA, while the cellular miRNA levels were calculated by normalizing to U6 RNA levels.

To quantify cellular mRNA, total RNA was isolated using TRIzol reagent (Invitrogen, Carlsbad, CA, USA) and reverse-transcribed using the Transcriptor First Strand cDNA Synthesis Kit (Roche Diagnostics, Risch-Rotkreuz, Switzerland). qPCR was performed in 10μl reaction volumes containing cDNA along with universal primers for each mRNA and FS Universal SYBR Green Master Rox (Roche). Cellular mRNA levels were normalized to those of β-actin. Fold-change was expressed as $2^{-\Delta\Delta}$Ct. The primers used are listed in Table S2.

Western blotting

Whole-cell protein was extracted using RIPA Lysis Buffer (Beyotime, Beijing, China), followed by centrifugation at 12,000 rpm for 15 minutes at 4°C. Protein levels were quantified using the BCA Protein Assay Kit (Beyotime, P0012). After immunoblotting, proteins were transferred onto polyvinylidene difluoride membranes (Millipore) and incubated with specific antibodies. The membranes were blocked using 5% skim milk

powder (Sigma) and incubated overnight at 4°C with primary antibodies. Subsequently, membranes were incubated with peroxidase-conjugated IgG antibodies (Zhongshan Goldbridge, Beijing, China) for one hour at room temperature. Protein bands were detected using the Super Chemiluminescent Reagent (HaiGene, Harbin, China). The β-actin antibody (Proteintech, Chicago, IL, USA) was used as an internal control. All the specific primary antibodies are shown in Supplementary Table S4.

Co-immunoprecipitations assay

The total protein from the cell lysate was immunoprecipitated. First, the extract was incubated with an antibody against acetylated-lysine (Cell Signaling) for 16 hours at 4°C. Then the extract was incubated with protein G beads for 6 hours at 4°C and centrifuged at 2000 rpm for 2 minutes. The beads were recovered, and aliquots of pellets were analyzed by immunoblotting with anti-PDH and anti-ATP synthase antibodies.

Luciferase assay

After transfected with miR-NC, miR-503 mimics, anti-NC, or anti- miR-503, SIRT3 or PGC-1β 3'UTR sequences including the binding sites for miR-503 or mutant 3`-UTR of SIRT3 or PGC-1β sequences were co-transfected with a firefly luciferase reporter and Renilla luciferase reporter into HEK293 cells utilized Lipofectamine 3000 (Invitrogen, Carlsbad, USA). Luciferase intensity was detected using the dual-luciferase reporter assay system (Promega, Madison, WI, USA). Each group was cultured in 96-well plates in triplicates.

m6A RNA immunoprecipitation assay (MeRIP)

m6A RNA-binding assays were performed using the Magna MeRIP m6A Kit (Millipore, Billerica, MA, USA). In brief, the isolated RNAs were fragmented to ~100 nt and incubated with the magnetic beads (Life Technologies, USA) previously conjugated with anti-m6A antibody (1:1000, Abcam, USA) overnight at 4°C in the RIP immunoprecipitation buffer. The interacting RNAs were extracted, analyzed by qRT-PCR using primers for pri-miR-503, and normalized to input.

miRNA target Immunoprecipitation

A biotinylated miR-503 pull-down assay with the target mRNA was performed as previously reported [2]. Briefly, MAECs were transfected with biotin-labeled miR-NC or miR-503 (RiboBio Co. Ltd. Guangzhou, China) were lysed and incubated with streptavidin-Dyna beads (Invitrogen, USA) overnight at 4°C rotating. The supernatant was discarded. The beads were then washed three times with ice-cold lysis buffer 3 times. Both the input and pull-down RNA samples were extracted with TRIzol Reagent, followed by reverse transcription and qRT-PCR analysis.

Chromatin immunoprecipitation (ChIP)

ChIP was performed using the SimpleChIP Enzymatic Chromatin IP Kit (Cell Signaling Technology, #9003), according to the manufacturer's instructions. Briefly, DNA and proteins in the endothelial cells were cross-linked with 1% formaldehyde. Next, the cell lysates were sonicated on ice to generate 200–300 bp chromatin fragments. The pre-cleared DNA was

saved as an input fraction. Pre-cleared DNA was immunoprecipitated with ChIP-grade H3K4me3 antibodies or IgG as a nonspecific control. The precipitated chromatin DNA was purified and detected by qPCR using the primers listed in Supplementary Table S2.

RNA fluorescence in situ hybridization (FISH)

FISH was performed using a fluorescent in situ hybridization kit (RiboBio, Guangzhou, China), according to the manufacturer's protocol. Briefly, MAECs were fixed and incubated with a specific FISH probe (RiboBio, Guangzhou, China) in hybridization buffer overnight at 37°C. The cells were washed, and immunofluorescence and DAPI staining were performed.

Flow cytometry analysis of apoptosis

The cells were washed twice with cold PBS and resuspended in the binding buffer. Each sample was calculated using 1×10^5 cells (100 µl) and incubated with 5 µl of FITC Annexin V and 2.5 µl of propidium iodide (PI) (BD Bioscience, San Jose, CA, USA) for 15 minutes in the dark. Subsequently, 200 µl of binding buffer was added to each sample. The percentage of positive cells was analyzed using a FACSCanto II flow cytometer (BD Biosciences).

Cardiac cell isolation

The hearts were harvested and digested with trypsin and collagenase. Cellular selection was performed as previously described[3]. Briefly, digested tissues were centrifuged at 80 g for 1 minute at 4°C. The pellet was plated for 40 minutes. Nonadherent cells were selected and regarded as cardiomyocytes. The centrifugation supernatant was used to isolate the

hematopoietic cell, endothelial cell, and fibroblast cell subsets by flow cytometry separation with anti-CD45 and anti-CD144 antibodies.

Viability assays

Cell viability was determined using a Calcein-AM/PI live and dead staining kit (Solarbio, Beijing, China), in accordance with the manufacturer's instructions. The cells were washed with assay buffer three times, followed by addition of calcein-AM (2mM) and PI (1.5mM) and incubated for 20 minutes at 37°C in the dark. Living and dead cells were identified by green and red fluorescence.

Colony formation and EdU labeling assay

For the colony formation assay, transfected cells were seeded into 6 well plates at a density of 800 cells per well and cultured for 2 weeks with DMEM containing 10% FBS. The cells were then fixed with 4% paraformaldehyde for 30 minutes and stained with a crystal violet staining solution (Beyotime, C0121) for 30 minutes. After washing with PBS 3 times, colonies were observed and counted.

For the EdU labeling assay, transfected cells were seeded into 96 well plates and incubated with EdU solution following the instructions of the keyFluor488 Click-iT Kit (KeyGen BioTECH, KGA331).

Mitochondrial membrane potential measurement

A mitochondrial membrane potential assay kit with JC-1 (Solarbio, M8650) was used to assess mitochondrial membrane potential. The cells were harvested and washed with JC-1Staining buffer. The JC-1 reagent solution was added and incubated for 20 minutes at 37°C in the dark. The cells were washed thrice with JC-1 staining buffer. The fluorescence of JC-1 aggregates and JC-1 monomers was analyzed using a FACSCanto II flow cytometer (BD Biosciences, CA, USA).

DCFH-DA staining

DCFH-DA staining was used to analyze ROS production using a reactive oxygen species assay kit (MCE, Monmouth Junction, NJ, USA). In brief, cells were inoculated into 96-well plates with 200-μL medium and incubated in darkness with DCFH-DA (10 μmol/L) for 30 minutes at 37°C. DCF fluorescence intensity was measured using a fluorescence microplate reader at excitation and emission wavelengths of 488 nm and 525 nm, respectively.

Mitochondrial oxygen consumption

The mitochondrial oxygen consumption rate (OCR) was calculated using an XF24 extracellular flux analyzer (Seahorse Biosciences). Briefly, 60,000 cells were seeded per well in XF24 culture plates. The cells were cultured under normoxic or hypoxic conditions for the indicated times, followed by incubation with the assay medium. Mitochondrial oxygen consumption was measured under basal conditions throughout treatment with several injections of oligomycin (1 μM), antimycin A (0.5 μM), FCCP (0.5 μM), and rotenone/antimycin A (0.5 μM) to assess maximal mitochondrial respiration.

Enzymatic activity assay

Pyruvate dehydrogenase (PDH) activity was analyzed using the PDH Enzyme Activity Microplate Assay Kit (Abcam). In brief, cells were harvested, and the samples were extracted using Detergent Solution. The samples were then loaded onto a capture antibody precoated microplate and incubated for 3h. Finally, the wells were washed with a stabilizer solution, and the assay solution was added. PDH enzyme activity was quantified calorimetrically on a microplate reader at an absorbance of 450 nm.

ATP synthase enzyme activity was analyzed using an ATP synthase enzyme activity microplate assay kit (Abcam). In brief, cells were harvested, and the samples were extracted using Detergent Solution. The samples were then loaded onto a capture antibody precoated microplate and incubated for 3 hours at room temperature. Finally, the wells were washed and the lipid mix was added. ATP synthase enzyme activity was quantified calorimetrically on a microplate reader at an absorbance of 340 nm.

RNA m6A quantification

The m6A RNA Methylation Assay Kit (Colorimetric, ab185912, Abcam) was used to quantify methylated N6-methyladenosine (m6A) in total RNA. Briefly, 200 ng of RNA isolated with TRIzol reagent was coated on the assay wells. Diluted capture antibody was added to each well and incubated at room temperature. Developer solution was added to each well, followed by a stop solution to stop the enzyme reaction. m6A levels were quantified

colorimetrically on a microplate reader at an absorbance of 450 nm. Absolute quantification was performed according to the standard curve.

EVs labeling

For EV-tracing in vitro experiments, 2 μg of EVs labeled with PKH67 fluorescent cell membrane dye (Sigma, St. Louis, MO) was incubated with 2×10^5 recipient cells and measured by confocal laser scanning microscopy (Leica, JEM-1220, JEOL, Ltd., Japan) or flow cytometry (BD Biosciences, San Jose, CA, USA).

To identify EV internalization in cardiomyocytes in vivo, PKH67 stained EVs were administered intramyocardially. Hearts were harvested 1 hour after injection. After immunofluorescence staining, images were obtained using a confocal laser scanning microscope.

The generation of endothelial cell-specific *Mettl3* knockout mice

Mettl3 $^{fl/fl}$ mice were crossed with Cdh5-CreERT2 mice to generate endothelial cell-specific Mettl3 knockout mice. Mettl3 $^{fl/fl}$ mice with a C57BL/6 background were provided by the HIT Center for Life Sciences (Harbin, Heilongjiang, China). The *Mettl3* $^{flox/flox}$ mice, as exons 2 and 3 of the *Mettl3* gene were flanked by two loxp sites, were generated using the CRISPR-Cas9 technique as previously described[4,5]. *Cdh5-CreERT2* mice were generated using Cyagen Biosciences lnc. (Suzhou, Jiangsu, China). Inducible deletion of Mettl3 in the Cdh5-CreERT2 model was achieved by tamoxifen treatment (40 mg/kg i.p. for three consecutive

days). 8-16 weeks Male mice with cell-specific *Mettl3* deletions were compared with Cre-negative Flox littermates.

Intramyocardial injection of EVs

Intramyocardial injection of EVs was performed following identification of induced ischemia via epicardial blanching, as previously described [6]. Briefly, EVs isolated from 200 μl of serum and re-suspended in 20 μl sterile PBS were injected into the cardiac infarct border zone as two separate 10 μl injections (one on either side of the ligation). After the injection, the pericardium was re-draped over the heart, and the chest was closed. In the control group, an equal volume of PBS was injected at the same location.

Ultrasound and magnetic resonance imaging

Two-dimensional and M-mode echocardiographic images were obtained in the parasternal long-axis view using a high-resolution in vivo VIVID E9 imaging system (GE, USA). The left ventricular end-diameter during diastole (LVEDd), systole (LVESd), diastolic interventricular septum thickness (IVSTd), along with the left ventricular posterior wall thickness during diastole (LVPWTd) were measured. Left ventricular ejection fraction (EF) and fractional shortening (FS) were calculated from five cardiac cycles. Left ventricular function was also evaluated by cardiac magnetic resonance imaging (MRI) using a 9.4-T small animal MRI scanner (Bruker BioSpec 94/20USR, Erlangen, Germany). Myocardial function was assessed by investigators who were blinded to the treatment group.

Histological, immunofluorescence, and cardiac injury biomarker analysis

Cardiac tissue was excised under anesthesia after echocardiography and perfused with 4% paraformaldehyde. For histological staining, heart tissue was successively dehydrated, cleared, and embedded. Then, 5μm sliced wax sections were subjected to Masson (Solarbio, Beijing, China) staining according to the manufacturer's instructions. Infarct area was calculated as the total infarct circumference divided by the total left ventricle circumference. In brief, the endocardial infarct length was taken as the length of the endocardial infarct scar surface, which included 50% of the whole thickness of the myocardium, and epicardial infarct length as the length of the transmural infarct region. The endocardial infarct ratio was calculated similarly. The infarct size was calculated as follows: [(epicardial infarct ratio + endocardial infarct ratio)/2] × 100.

For immunofluorescence and WGA staining, tissues were embedded within an optimal cutting temperature (Tissue-Tek) and cut to 7μm. The sections were then incubated with anti-TnI antibody. WGA staining was performed using FITC-labeled wheat germ agglutinin dye (Sigma-Aldrich, St. Louis, MO, USA), as described previously. For the TUNEL assay, the sections were treated with TUNEL dye (Roche, 11684817910) and incubated for 60 minutes. Cell nuclei were counterstained with DAPI dye. Cardiac troponin I and lactic acid dehydrogenase levels were measured 24 hours after myocardial infarction using ELISA kits (Nanjing Jiancheng, China). All analyses were performed by performers blinded to the treatment group.

1. Ehler E, Moore-Morris T, Lange S. Isolation and culture of neonatal mouse cardiomyocytes. *J Vis Exp.* 2013(79).
2. Orom UA, Lund AH. Isolation of microRNA targets using biotinylated synthetic microRNAs. *Methods.* 2007;43(2):162-165.
3. Rogg EM, Abplanalp WT, Bischof C, et al. Analysis of Cell Type-Specific Effects of MicroRNA-92a Provides Novel Insights Into Target Regulation and Mechanism of Action. *Circulation.* 2018;138(22):2545-2558.
4. Wang Y, Gao M, Zhu F, et al. METTL3 is essential for postnatal development of brown adipose tissue and energy expenditure in mice. *Nat Commun.* 2020;11(1):1648.
5. Li X, Jiang Y, Sun X, Wu Y, Chen Z. METTL3 is required for maintaining beta-cell function. *Metabolism.* 2021;116:154702.
6. Khan M, Nickoloff E, Abramova T, et al. Embryonic stem cell-derived exosomes promote endogenous repair mechanisms and enhance cardiac function following myocardial infarction. *Circ Res.* 2015;117(1):52-64.

Supplementary Figures

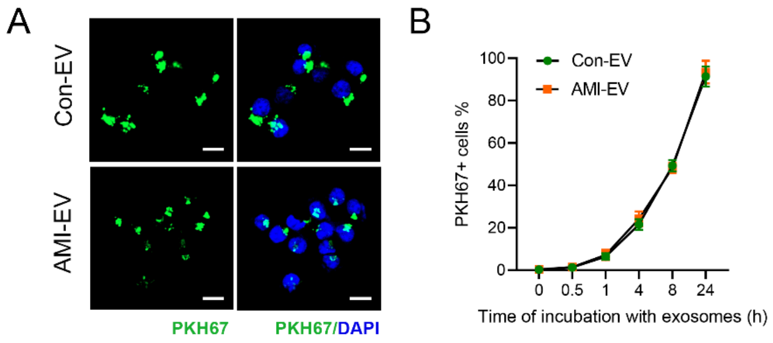

FIGURE S1 The measurement of AC16 cells to internalize extracellular vesicles (EVs).

A, The uptake of EVs in cardiomyocytes was verified by laser scanning confocal microscope 24 hours after incubation. Nuclei were stained with DAPI dye (blue) and EVs were labeled with PKH67 (green). Scale bar, 20 μm. **B,** Flow cytometry analysis of the PKH67-labeled EVs uptake by AC16 cells for indicated times.

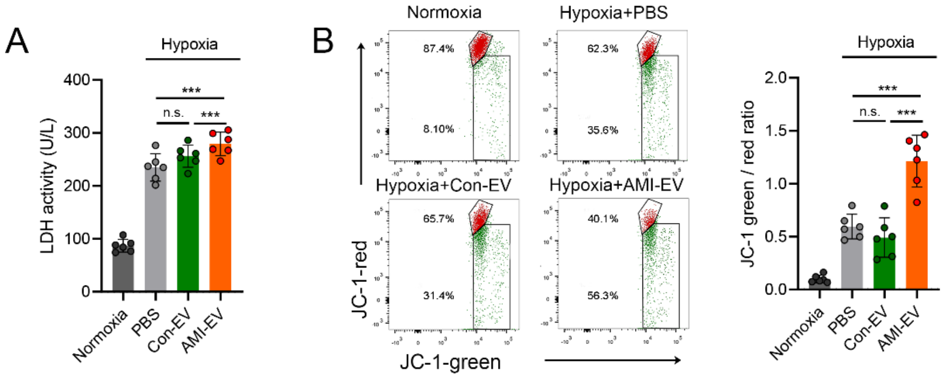

FIGURE S2. Analysis of AMI serum extracellular vesicles (EVs) on AC16 cells survival.

A, LDH release levels were measured in indicated groups. **B,** Flow cytometry analysis of JC-1 staining indicating mitochondrial membrane depolarization (n = 6, ***p < 0.001).

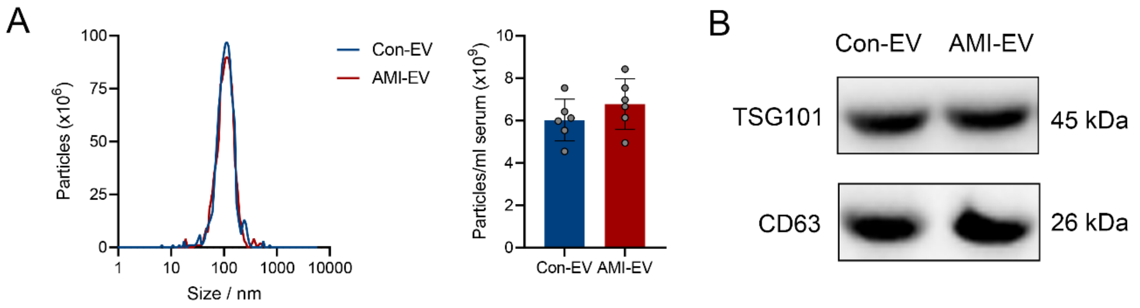

FIGURE S3. The identification of extracellular vesicles (EVs) isolated from mice serum after LAD ligation. A, Representative images of nanoparticle tracking analysis reflecting size distributions of isolated vesicles from AMI mice or sham control group. The average concentration of serum EVs in AMI and control mice were calculated (n = 6). **B,** Representative blot images of exosomal markers of TSG101 and CD63 in isolated vesicles.

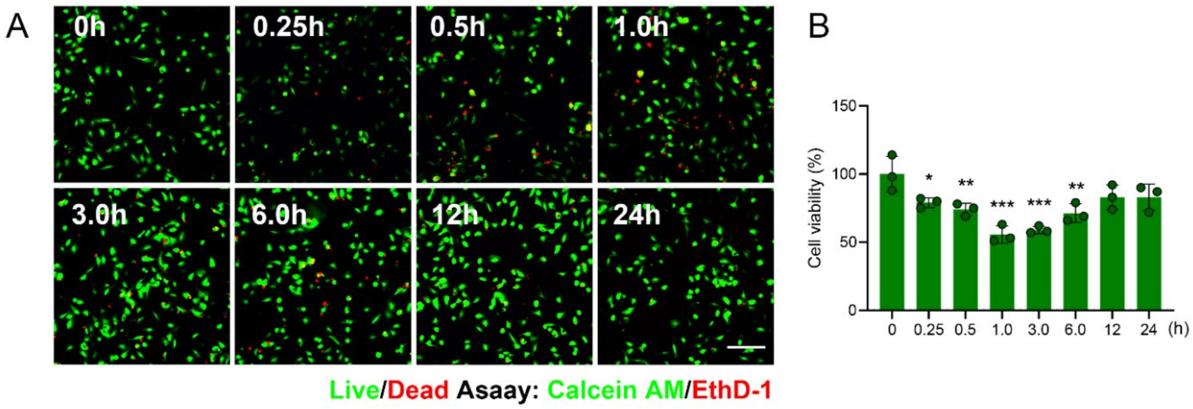

FIGURE S4. Effect of acute myocardial infarction-extracellular vesicles (acute myocardial infarction-extracellular vesicles (AMI-EVs) on cardiomyocytes viability. A, Fluorescence staining with calcein-AM (green) and ethidium homodimer-1 staining (red) for NMCMs incubated with EVs isolated from different phase after left anterior descending ligation. Scale bar, 100 µm. **B,** The percentage of cell viability is calculated (n = 3). (*p < 0.05; **p < 0.01; ***p < 0.001).

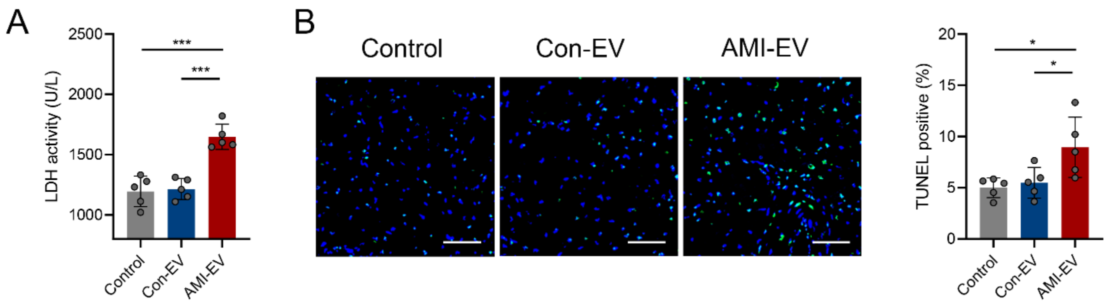

FIGURE S5. Effect of acute myocardial infarction-extracellular vesicles (AMI-EVs) on normal mice. A, Serum LDH activity after EVs injection (n = 5). B, TUNEL immunofluorescence staining identifies the apoptosis cells in cardiac tissue, followed by DAPI staining for visualizing nuclei. Scale bar, 100 μm. The TUNEL-positive cardiomyocytes quantification was shown on the right panels (n = 5). (*p < 0.05; ***p < 0.001).

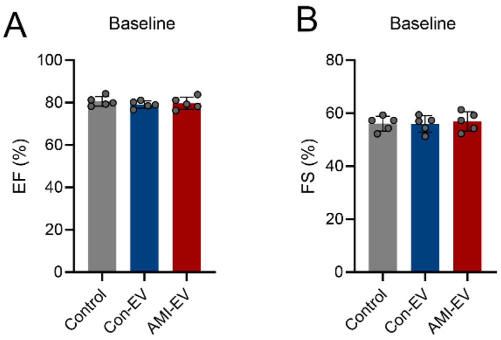

FIGURE S6. Baseline echocardiographic parameters of mice before left anterior descending ligation. A, EF was measured by echocardiography at baseline on 1 day before left anterior descending ligation. **B,** FS was measured by echocardiography at baseline on 1 day before left anterior descending ligation. (n = 5).

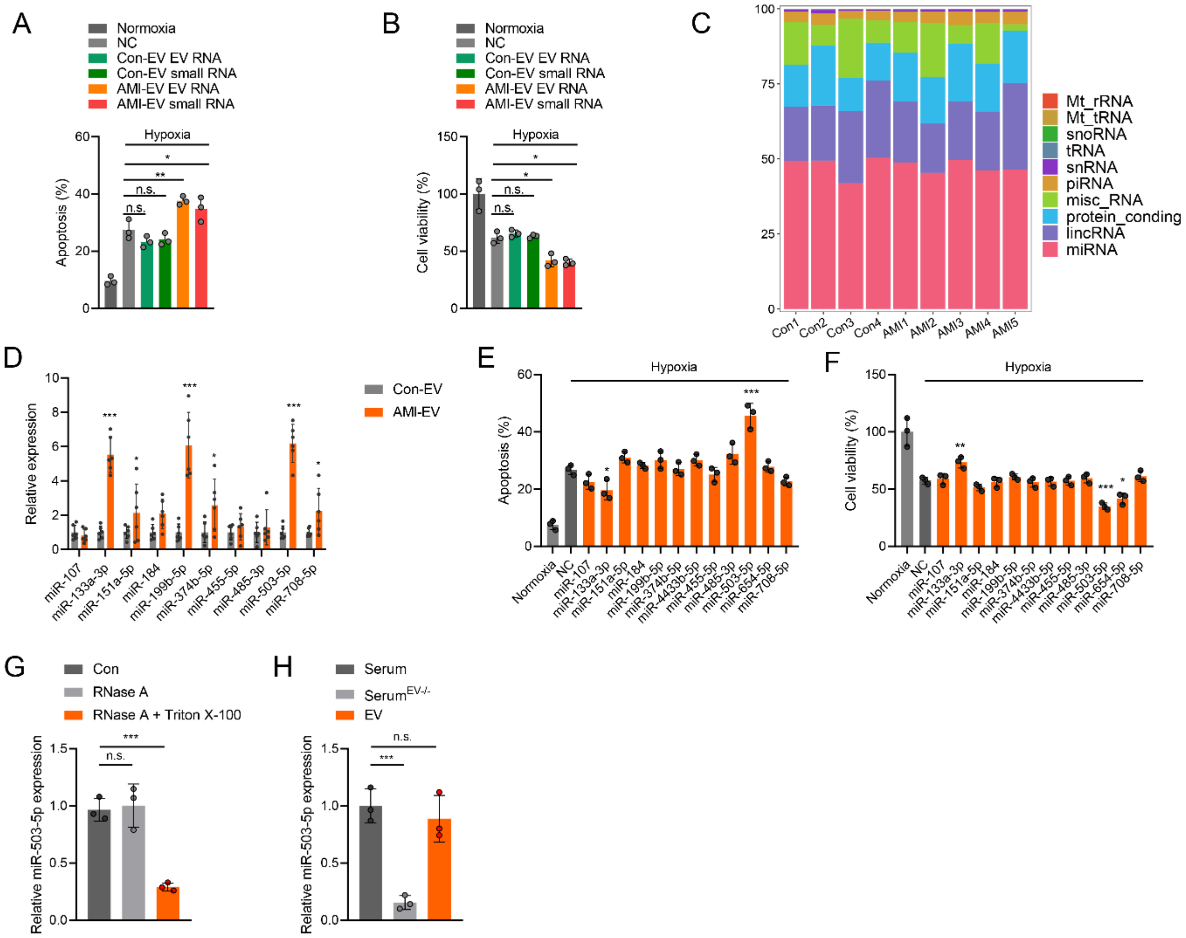

FIGURE S7. Identification of miR-503 highly expressed after AMI. A, AC16 cells transfected with an equal amount of total or small (< 200 nt) RNA extracted from extracellular vesicles (EVs) from AMI serum or control groups, or negative control (NC) RNA (cel-miR-67), were subjected to Annexin V-APC and PI staining for apoptosis analysis after 12 hours of hypoxia after transfection (n = 3). **B,** AC16 cells transfected with an equal amount of total or small (<200 nt) RNA extracted from EVs from AMI serum or control group, or negative control RNA (cel-miR-67), were subjected to calcein-AM and ethidium homodimer-1 staining for cell viability analysis 12h hypoxia after transfection (n = 3). **C,** Average percentage of biotype counts of each sample from AMI patients and healthy control group. D, AC16 cells were transfected with miRNAs differentia. **D,** The expression of

miRNAs in mice EVs after AMI (n = 6). **E,** Annexin V-APC and PI staining for apoptosis analysis in AC16 cells transfected with a mimic of indicated miRNA or negative control (NC) after 12h hypoxia after transfection (n = 3). **F,** Calcein-AM and ethidium homodimer-1 staining for cell viability analysis in AC16 cells transfected with a mimic of indicated miRNA or negative control (NC) after 12h hypoxia after transfection (n = 3). **G,** qRT-PCR analysis for miR-503 expression in EVs from AMI patients treated with RNase A or RNase A combined with Triton X-100 (n = 3). **H,** qRT-PCR analysis for miR-503 expression in serum, serum depletion of EVs or EVs from AMI patients (n = 3). (*$p < 0.05$; **$p < 0.01$; ***$p < 0.001$).

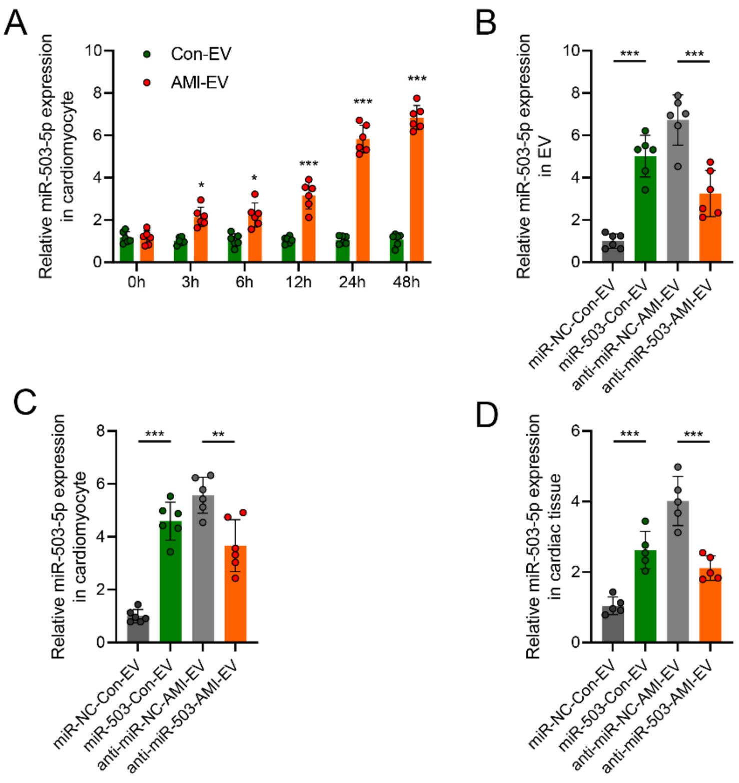

FIGURE S8. The transport validation of exosomal miR-503 on cardiomyocyte and cardiac tissue. A, Cardiomyocytes were incubated with extracellular vesicles (EVs) from AMI mice or the control group. The expression of cellular miR-503 was measured in indicated time (n = 6). **B,** qRT-PCR analysis for miR-503 expression in EVs from control mice transfected with miR NC, or miR-503 and EVs from AMI mice transfected with anti-miR-NC, or anti-miR-503 (n = 6). **C,** qRT-PCR analysis for miR-503 expression in cardiomyocytes incubated with EVs from control mice transfected with miR-NC, or miR-503 and EVs from AMI mice transfected with anti-miR-NC, or anti-miR-503 (n = 6). **D,** qRT-PCR analysis for miR-503 expression in cardiac tissue treated with EVs from control mice transfected with miR NC, or miR-503 and EVs from AMI mice transfected with anti-miR-NC, or anti-miR-503 (n = 5). (*p < 0.05; **p < 0.01; ***p < 0.001).

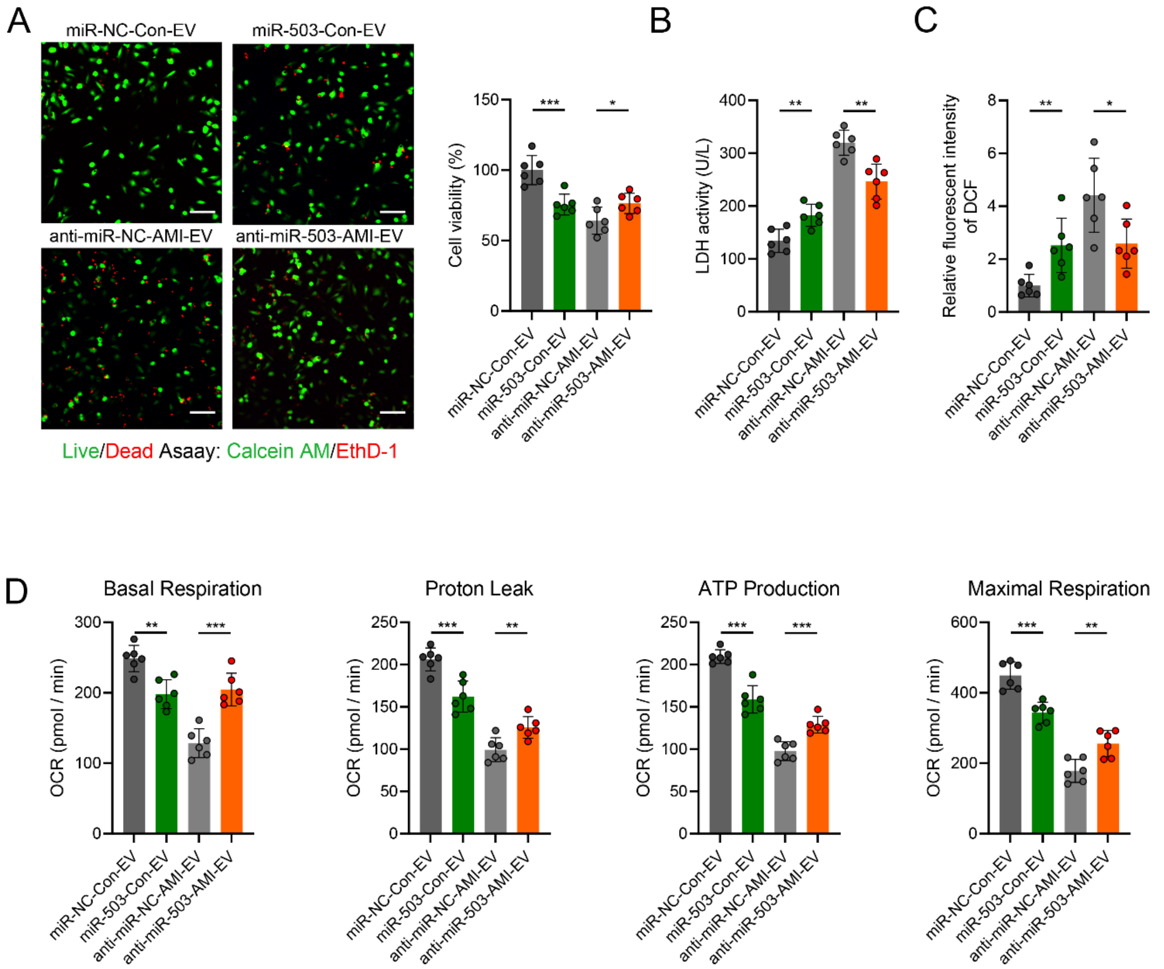

FIGURE S9. The functional validation of exosomal miR-503 on cardiomyocytes. A, Calcein-AM (green) and ethidium homodimer-1 staining (red) for cardiomyocytes after treatment with extracellular vesicles (EVs). The percentage of cell viability was calculated on the right panels. Scale bar, 100 μm. **B,** Cardiomyocyte LDH release levels were measured in indicated groups. **C,** Effect of EVs on cardiomyocytes ROS production was measured using fluorescence microplate reader for fluorescence intensity of DCF. **D,** Indices of mitochondrial respiration calculated from the obtained OCR profiles: Basal respiration, ATP production, proton leak, and maximal respiration in cardiomyocytes. (n = 6; *p < 0.05; **p < 0.01; ***p < 0.001).

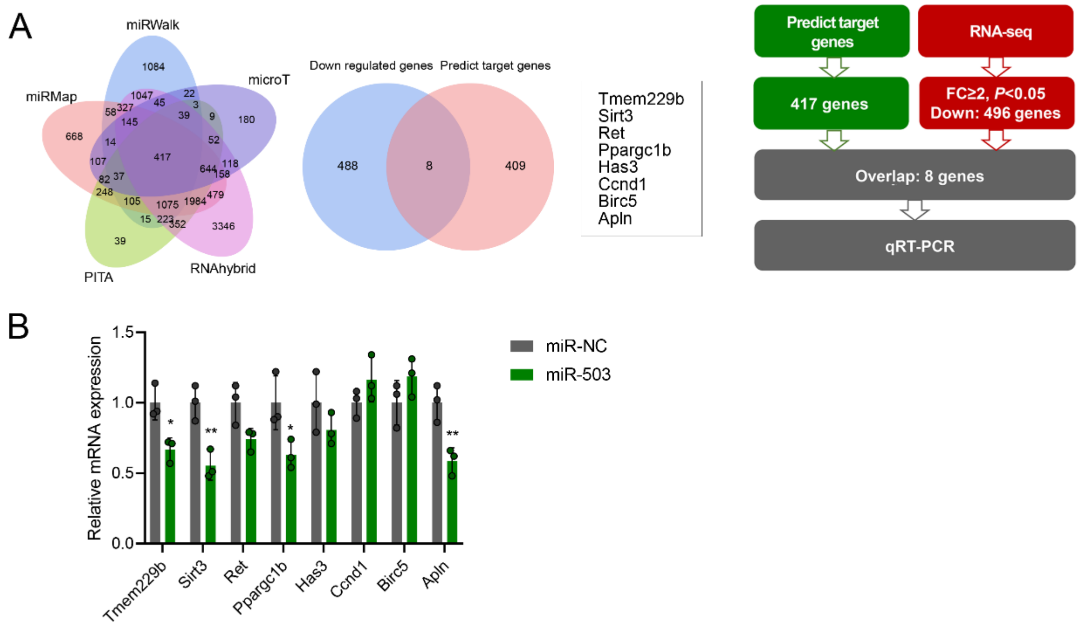

FIGURE S10. Search for the target genes of miR-503. A, Prediction of miR-503 target genes with miRWalk, microT, miRMap, PITA, and RNAhybrid (left). Screening scheme for potential target genes regulated by miR-503 (right). **B**, qRT-PCR analysis for mRNA expression of the potential target genes (n = 3; *p < 0.05; **p < 0.01).

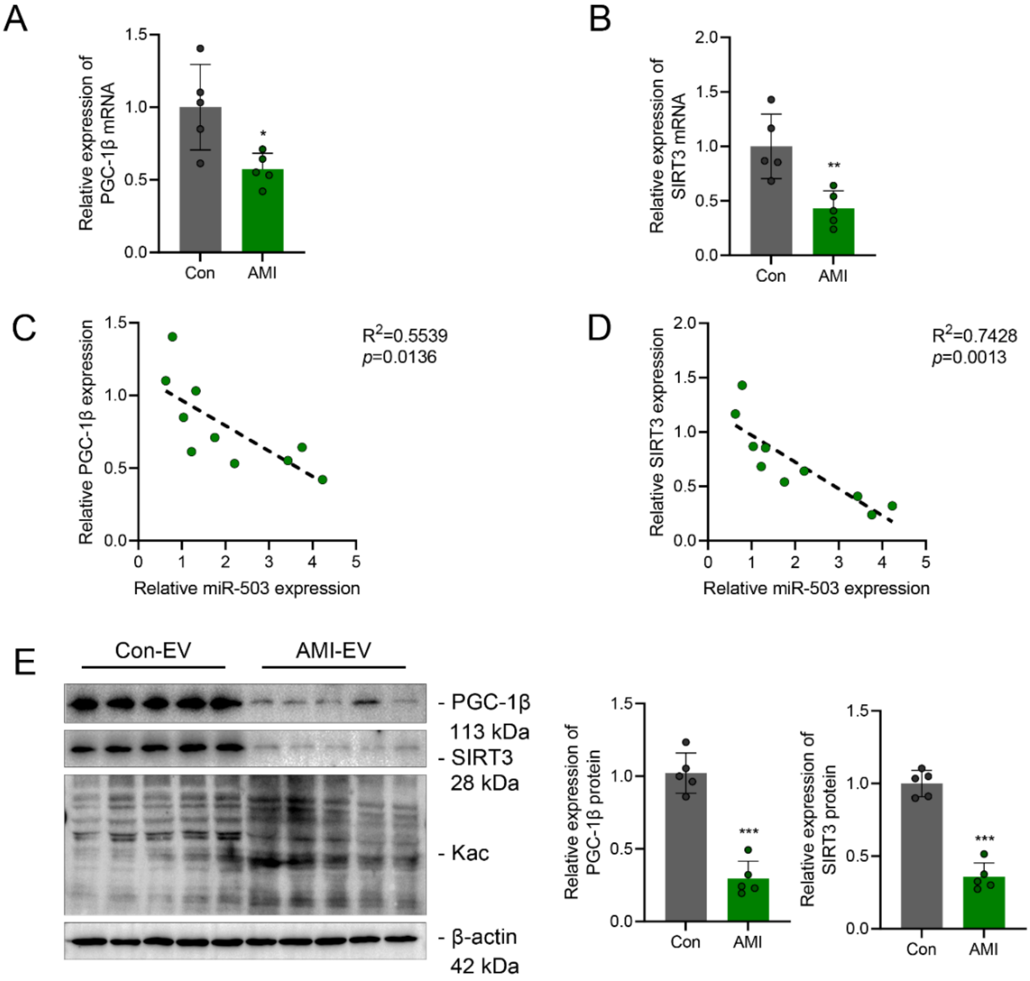

FIGURE S11. The expression of PGC-β and SIRT3 in mice hearts after LAD ligation. **A,** qRT-PCR analysis of PGC-β expression in mice hearts after LAD ligation and sham control group (n = 5). **B,** qRT-PCR analysis of SIRT3 expression in mice hearts after LAD ligation and sham control group (n = 5). **C,** Correlation analysis was performed between miR-503 expression and PGC-β mRNA levels in mice hearts. **D,** Correlation analysis was performed between miR-503 expression and SIRT3 mRNA levels in mice hearts. **E,** Representative blot images reflecting PGC-1β and SIRT3 protein levels and global acetylation levels in AMI mice hearts treated with Con-EV or acute myocardial infarction-extracellular vesicles (AMI-EVs) (n = 5). (*p < 0.05; **p < 0.01; ***p < 0.001).

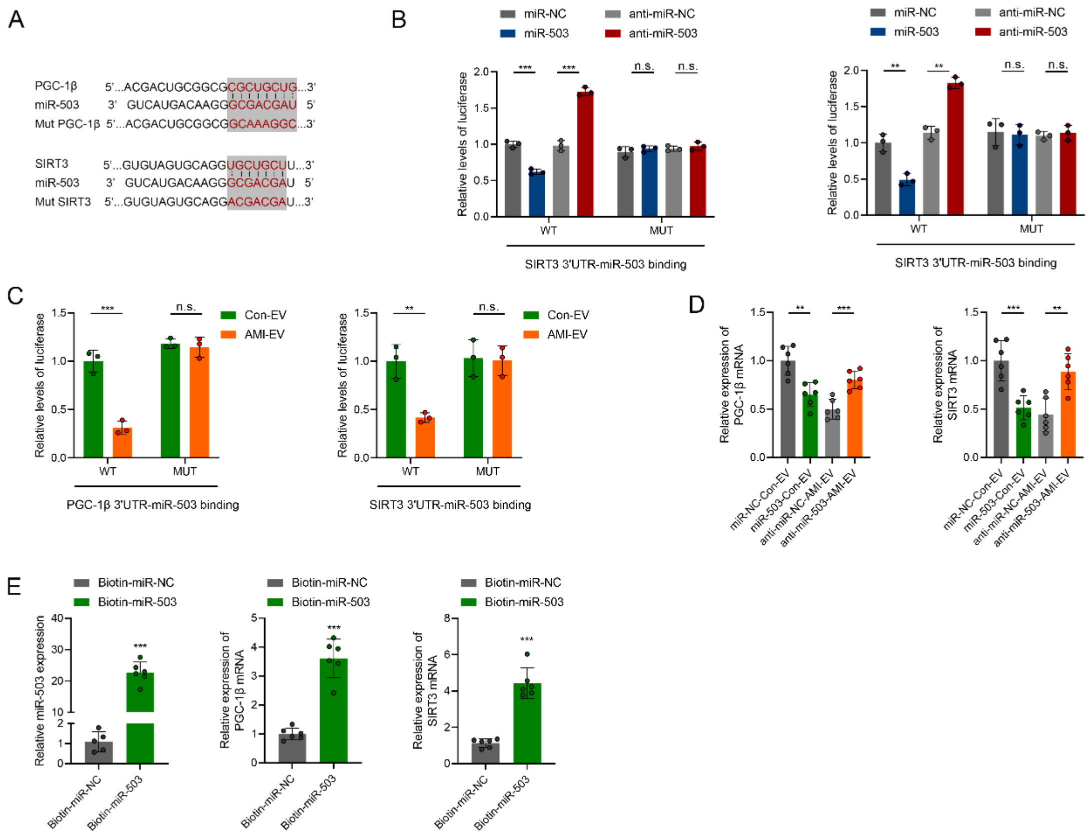

FIGURE S12. Identification PGC-β and SIRT3 as direct target genes of miR-503. A, Wild type and mutant binding site between miR-503 and PGC-β as well as SIRT3. **B,** After being transfected with miR-NC, miR-503 mimics, anti-NC, or anti- miR-503, PGC-1β, and SIRT3 3'UTR sequences including the binding sites for miR-503 or mutant 3`-UTR of PGC-1β and SIRT3 sequences were co-transfected with luciferase reporter (n = 3). **C,** Dual-luciferase reporter assays of 293T cells transfected with reporter plasmids containing the wild- or mutant-type PGC-β or SIRT3 and co-cultured with EVs from AMI patients or control group (n = 3). **D,** qRT-PCR analysis for PGC-β and SIRT3 expression in cardiomyocytes incubated EVs pre-treated with miR-503 or anti-miR-503 (n = 6). **E,** Cardiomyocytes were transfected with biotinylated miR-NC or miR-503. The miR-503 expression in cardiomyocytes and levels of PGC-β and SIRT3 mRNA in the materials pulled down by biotin-miR-503 were analyzed by qRT-PCR (n = 5; **p < 0.01; ***p < 0.001).

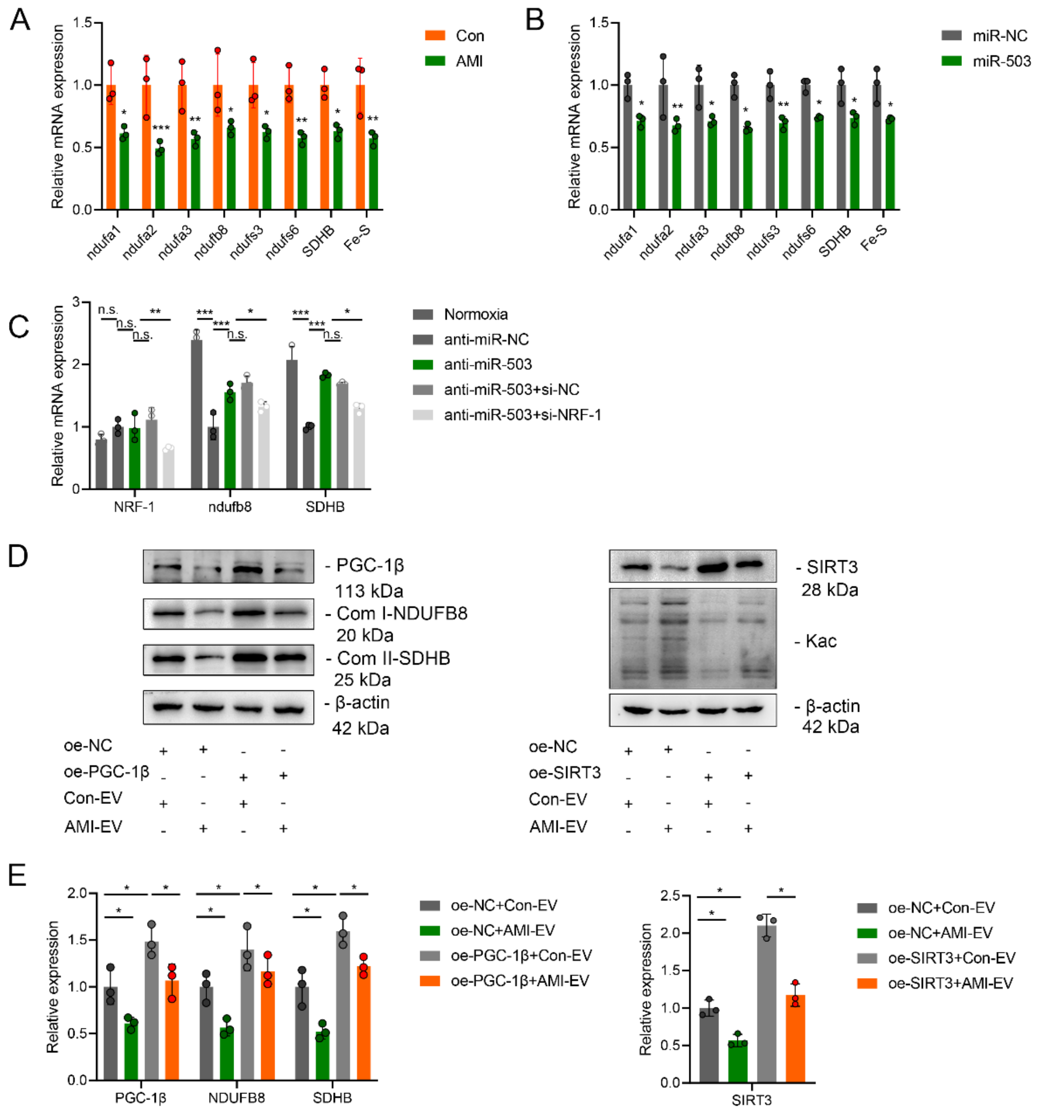

FIGURE S13. The regulatory effects of miR-503 on ETC components related genes. A, Relative expression of ETC components related genes in the heart from sham and LAD ligation mice (n = 3). **B,** Relative expression of ETC components related genes in cardiomyocytes transfected with miR-503 (n = 3). **C,** Relative expression of ETC components related genes in cardiomyocytes transfected with miR-503 inhibitor and si-NRF-1 under normal condition or hypoxia (n = 3). **D–E** Western blot images reflecting PGC-1β, NDUFB8, SDHB, SIRT3 protein levels and global acetylation levels in cardiomyocytes transfection with PGC-1β or SIRT3 plasmid and incubated with EVs (n = 3; *$p < 0.05$; **$p < 0.01$; ***$p < 0.001$).

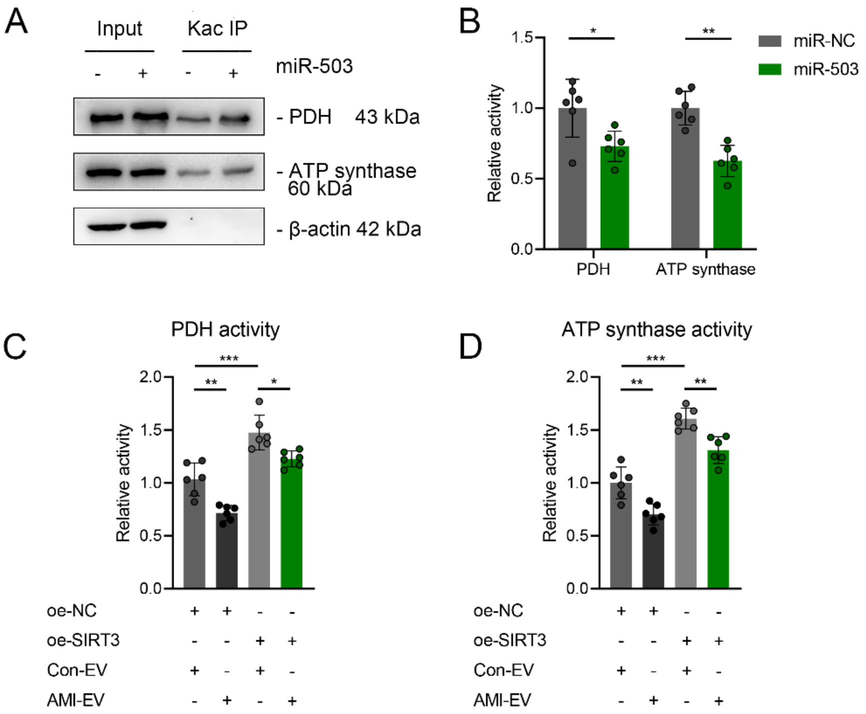

FIGURE S14. The downstream mechanism of miR-503/SIRT3 disturbing mitochondrial homeostasis. A, Cell lysates prepared from miR-503 transfected cardiomyocytes were subjected to IP assay using the acetylated-lysine antibody. Equal amounts of the pellets were performed western blotting with anti-PDH and anti-ATP-synthase antibodies. **B–C,** PDH activity, and ATP synthase activity in cardiomyocytes transfected with miR-503 were measured by activity microplate assay kits. **D,** PDH and ATP synthase activity in cardiomyocytes transfected with SIRT3 and incubated with EVs. (n = 6; *p < 0.05; **p < 0.01; ***p < 0.001).

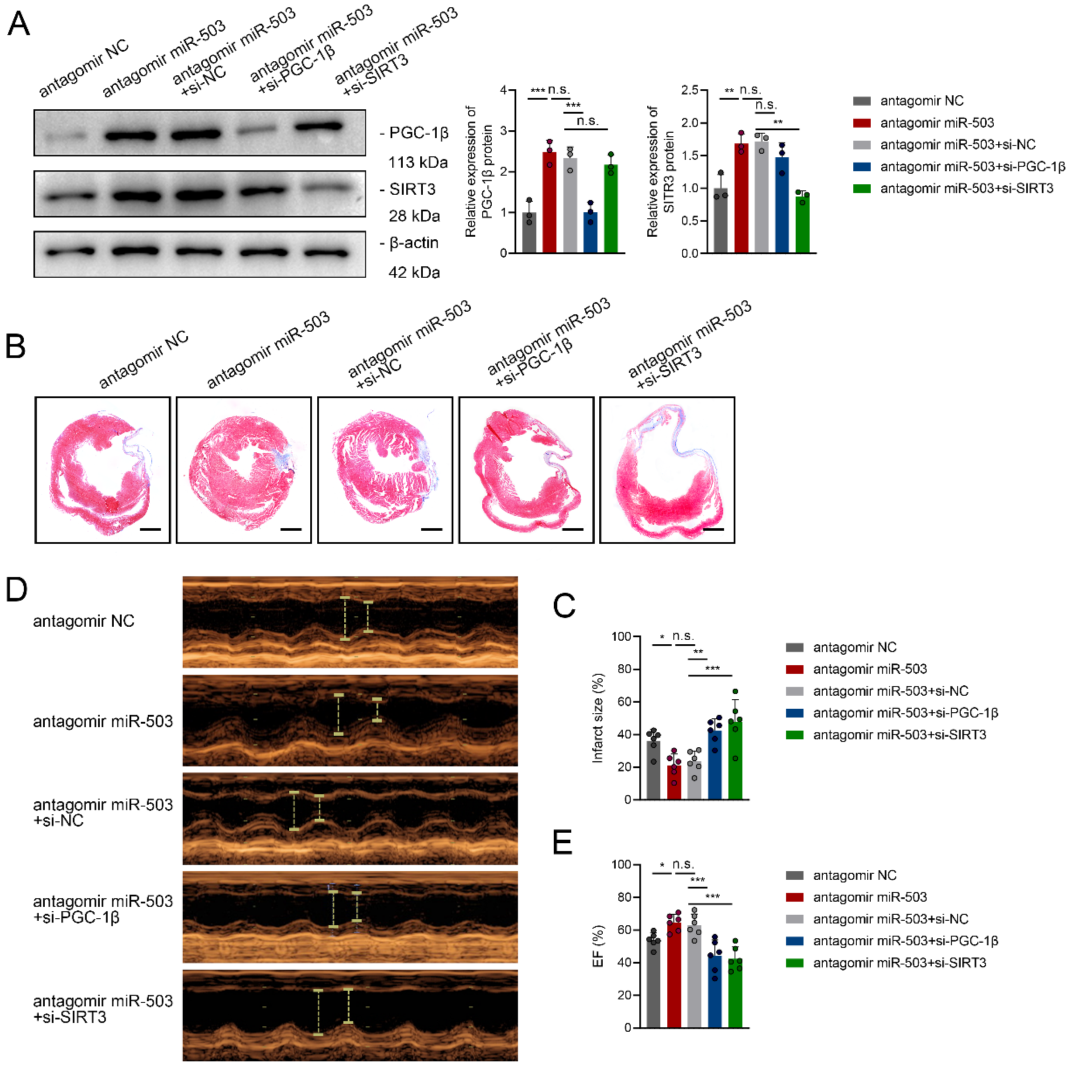

FIGURE S15. The functional analysis of miR-503 induce cardiac injury by targeting PGC-1β and SIRT3. miR-503 antagomirs, si-PGC-1β and si-SIRT3, were intramyocardially injected immediately following LAD ligation. **A,** Western blot images reflecting PGC-1β, and SIRT3 protein levels in cardiac tissue (n = 3), **B–C,** Representative images of the cardiac section with the Masson Trichrome Staining at 28 days after MI onset. Scale bar, 1 mm. The quantification of infarct size was shown (n = 6). **D–E,** Cardiac function measured by echocardiography and on 28 days after MI onset. The percentage of left ventricular ejection fraction (EF) was analyzed (n = 6). (*p < 0.05; **p < 0.01; ***p < 0.001).

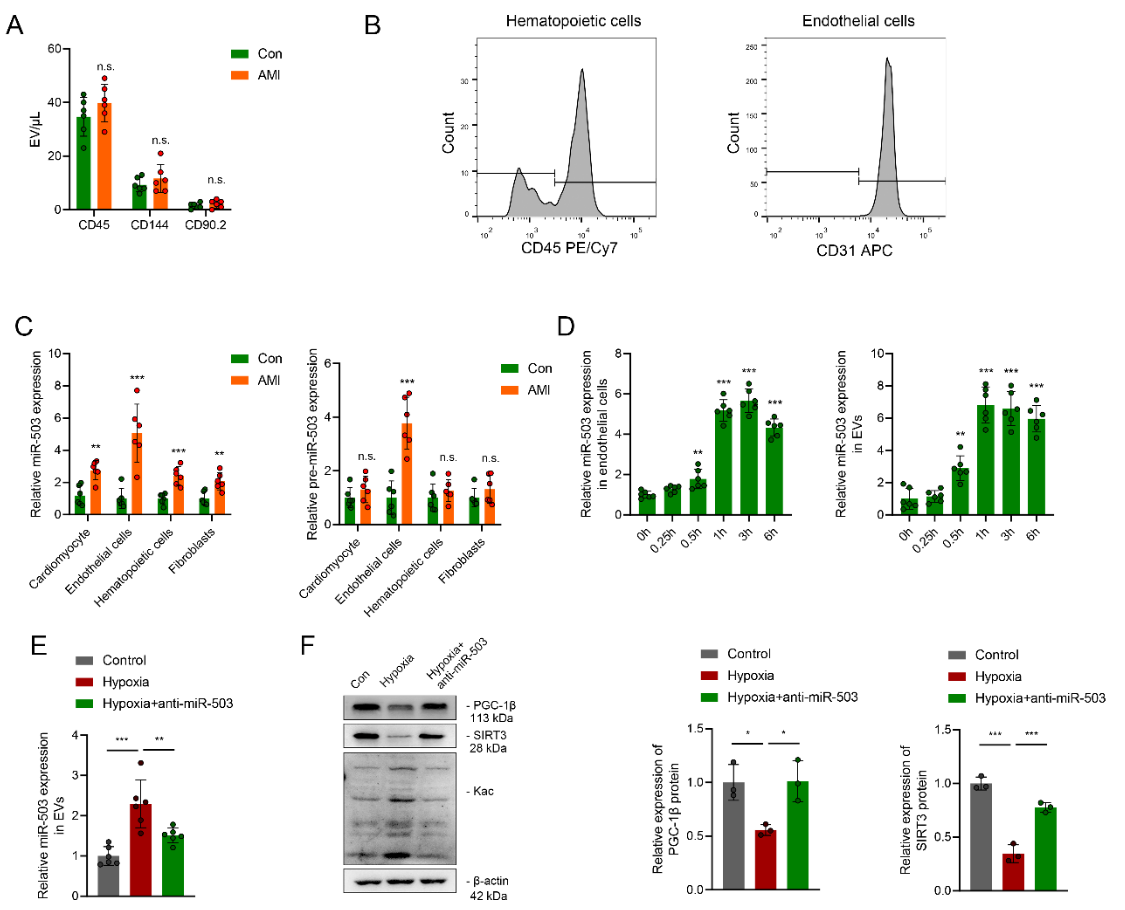

FIGURE S16. Identification endothelial cells under hypoxia as an origin of exosomal miR-503. A, The expression of CD45, CD144, and CD90.2 of EVs isolated from AMI mice was analyzed (n = 6). **B,** Flow cytometry analysis of the enrichment of hematopoietic cells ($CD45^+$) and endothelial cells ($CD31^+$) and after cell sorting. **C,** qRT-PCR analysis of miR-503 and pre-miR-503 expression in cardiomyocytes, fibroblasts, endothelial cells, and hematopoietic cells of AMI and control mice (n = 6). **D,** qRT-PCR analysis of miR-503 expression in mice aortic endothelial cells (MAECs) under hypoxia in indicated time points (left). qRT-PCR analysis of miR-503 expression in EVs isolated from MAECs under hypoxia in indicated time points (right) (n = 6). **E,** qRT-PCR analysis of miR-503 expression in EVs from miR-503 deficient MAECs under hypoxia (n = 6). **F,** PCG-1β, SIRT3 protein level, and global acetylation levels in cardiomyocytes incubated with EVs (n = 3). (*p < 0.05; **p < 0.01; ***p < 0.001).

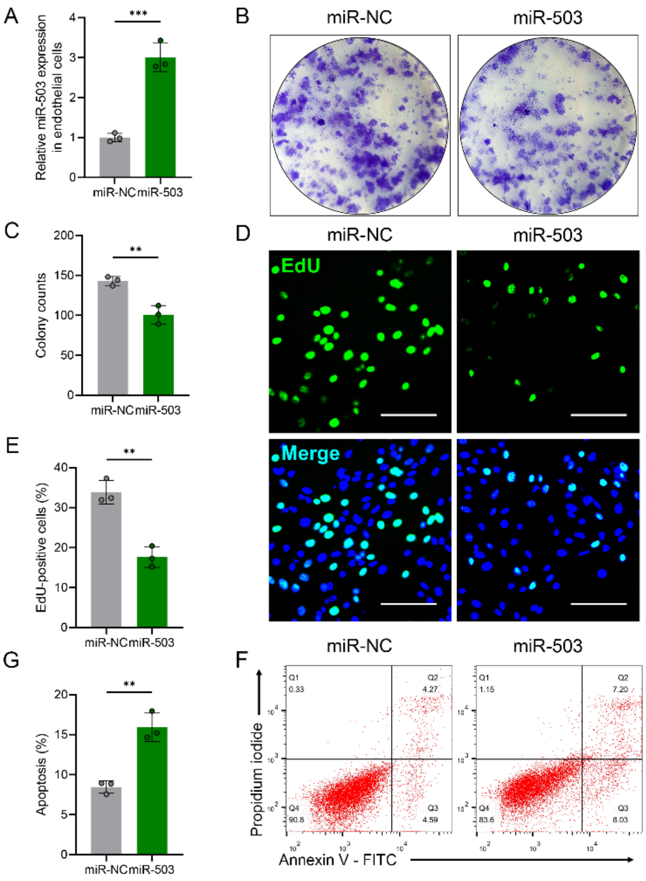

FIGURE S17. Functional analysis of miR-503 on endothelial cells. A, qRT-PCR analysis of miR-503 expression in MAECs transfected with mimic NC and mimic miR-503. **B–C,** Colony-formation ability of MAECs transfected with mimic NC and mimic miR-503. And colony counts in two groups were calculated. **D–E,** EdU staining for measuring cellular proliferation was performed in MAECs transfected with mimic NC and mimic miR-503. Scale bar, 100 μm. EdU positive cells were calculated in the two groups. **F–G,** Flow cytometry analysis of Annexin V-APC and PI staining in MAECs transfected with mimic NC and mimic miR-503. The cellular apoptosis rate was calculated in the two groups. (n = 3; $*p < 0.05$; $**p < 0.01$; $***p < 0.001$).

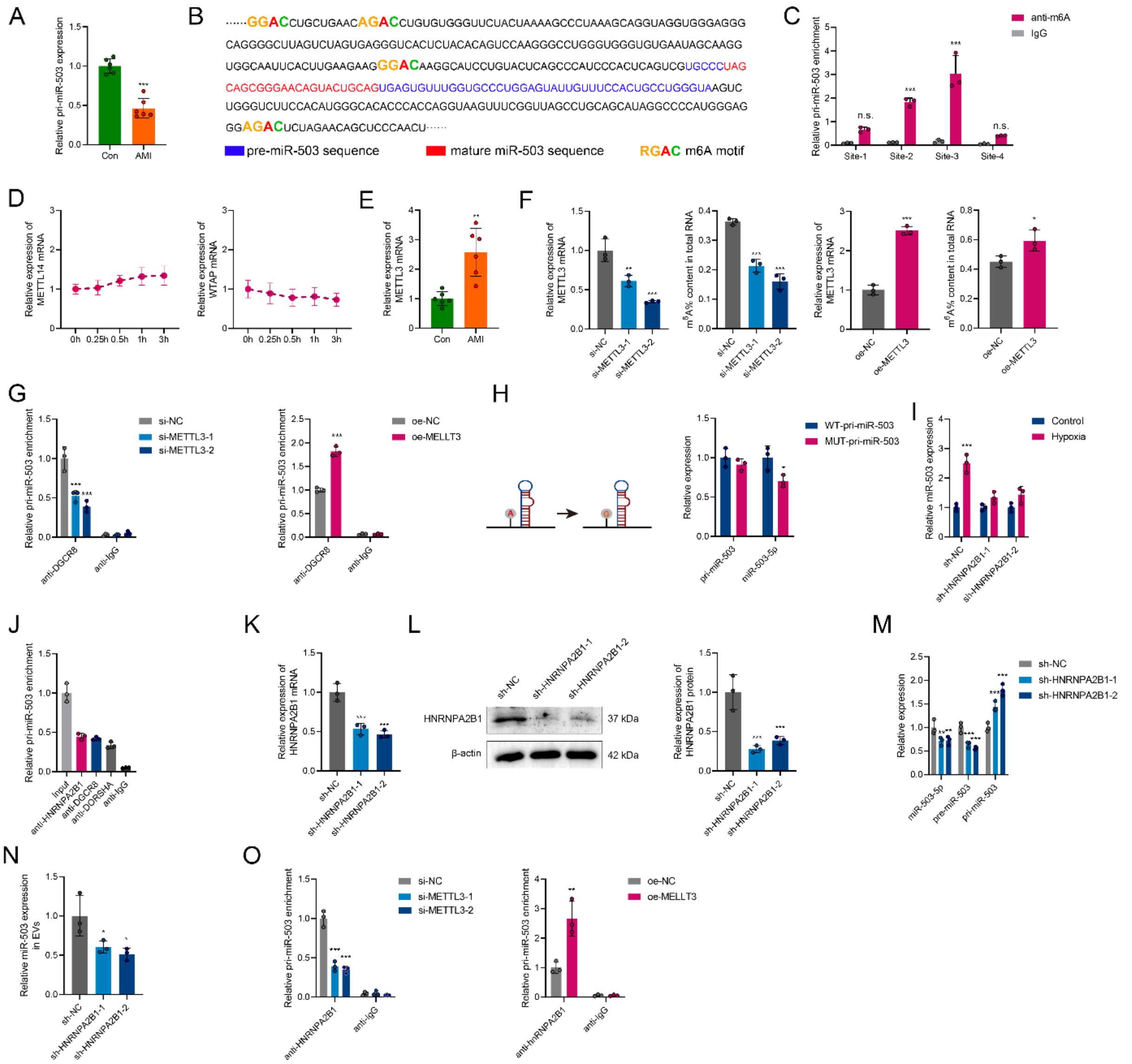

FIGURE S18. METTL3 could mediate pri-miR-503 maturation by N⁶-methyladenosine in endothelial cells after acute myocardial infarction (AMI). A, qRT-PCR analysis of pri-miR-503 expression in endothelial cells isolated from mice hearts after AMI (n = 5). **B,** The sequences of mice pre-miR-503 and mature miR-503 are highlighted, and the m6A motifs (RGAC) located nearby the putative splicing site are shown. **C,** Detection of pri-miR-503 m6A modification level in potential sites by immunoprecipitation of with an anti-m6A antibody in MAECs followed by qRT-PCR (n = 3). **D,** qRT-PCR analysis of METTL14 and

WTAP expression in MAECs under hypoxia in indicated time points (n = 3). **E,** qRT-PCR analysis of METTL3 expression in endothelial cells isolated from mice hearts after AMI (n = 5). **F,** qRT-PCR analysis of METTL3 expression in MAECs transfected with si-METTL3 or oe-METTL3 (n = 3). The m6A contents of total RNAs in MAECs transfected with si-METTL3 or oe-METTL3 (n = 3). **G,** Analysis of m6A modification on pri-miR-503 by immunoprecipitation of with an anti-DGCR8 antibody and qRT-PCR in MAECs transfected with si-METTL3 or METTL3 overexpression plasmid (n = 3). **H,** qRT-PCR analysis of pri-miR-503 and miR-503 expression in MAECs after transfected with pri-miR-503 or pri-miR-503 m6A site mutation (n = 3). **I,** qRT-PCR analysis of miR-503 expression in MAECs transfection with sh-HNRNPA2B1 under normoxia or hypoxia (n = 3). **J,** RNA immunoprecipitation coupled qRT-PCR analysis the pri-miR-503 levels and HNRNPA2B1 levels relative to input. Anti-DGCR8 or anti-DROSHA antibodies served as positive control while IgG was used as negative control (n = 3). **K,** qRT-PCR analysis of HNRNPA2B1 mRNA expression in MAECs transfected with si-HNRNPA2B1 (n = 3). **L,** Western blot analysis of HNRNPA2B1 protein expression in MAECs transfected with si-HNRNPA2B1 (n = 3). **M,** qRT-PCR analysis of pri-miR-503, pre-miR-503 and miR-503 expression in MAECs transfected with sh-HNRNPA2B1 (n = 3). **N,** Endothelial exosomal miR-503 expression after sh-HNRNPA2B1 transfected (n = 3). **O,** An anti-HNRNPA2B1 antibody for RIP assays coupled with qRT-PCR in MAECs transfected with si-METTL3 or METTL3 overexpression plasmid (n = 3; *$p < 0.05$; **$p < 0.01$; ***$p < 0.001$).

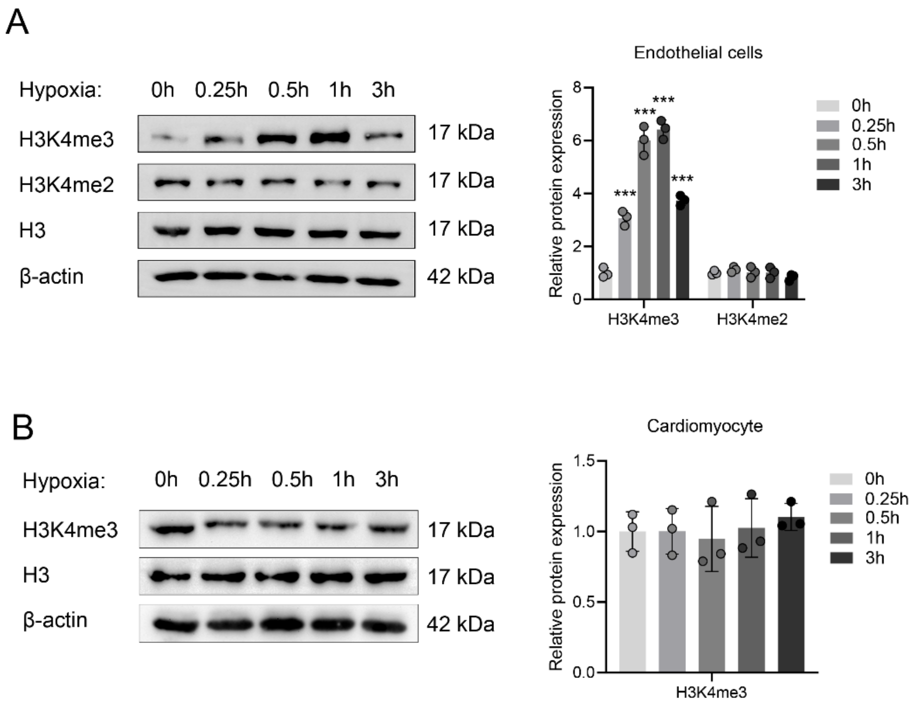

FIGURE S19. Expression of H3K4me3 in endothelial cells and cardiomyocytes under hypoxia. A, Western blot analysis of H3K4me3 and H3K4me2 expression in mouse aortic endothelial cells under hypoxia. **B,** Western blot analysis of H3K4me3 expression in primary cardiomyocytes isolated from neonatal mice under hypoxia (n = 3; *p < 0.05; **p < 0.01; ***p < 0.001).

Supplementary Tables

TABLE S1. Differentially expressed miRNAs in AMI EVs.

miRNA_id	Con-EV	AMI-EV	Fold Change	*p* Value	Regulation
hsa-miR-107	0.095339	18.27136	193.4259821	0.001119	Up
hsa-miR-133a-3p	0.023224	22.89432	623.1380069	0.000226	Up
hsa-miR-151a-5p	0.328802	16.22647	41.67874939	0.014435	Up
hsa-miR-184	0.255953	30.55747	103.6517159	0.000638	Up
hsa-miR-199b-5p	0.178221	18.02891	82.54353864	0.014029	Up
hsa-miR-374b-5p	0.176754	22.62316	150.0830879	0.00176	Up
hsa-miR-4433b-5p	0.244223	11.84235	45.85535214	0.008013	Up
hsa-miR-455-5p	0.051828	26.80233	510.0957837	0.001813	Up
hsa-miR-485-3p	0.192401	13.64252	59.23878977	0.004412	Up
hsa-miR-503-5p	1.245983	26.49781	23.82639903	0.035681	Up
hsa-miR-654-5p	0.167461	15.16172	72.55596496	0.005776	Up
hsa-miR-708-5p	0.14277	14.17156	99.40469362	0.002002	Up
hsa-miR-11400	18.76038	0.171847	0.009744937	0.000686	Down
hsa-miR-1229-3p	11.68301	0.025807	0.003870504	0.001948	Down
hsa-miR-129-5p	21.61409	0.117346	0.005696782	0.000511	Down
hsa-miR-133a-5p	17.2191	0.040885	0.001921728	0.000274	Down
hsa-miR-193b-5p	20.75201	0.243213	0.012026626	0.000405	Down
hsa-miR-215-5p	23.90236	0.142405	0.004899899	5.86E-05	Down
hsa-miR-374a-3p	5.023069	0	0.003637166	0.005496	Down
hsa-miR-425-5p	41.17832	0.13025	0.003506719	1.30E-05	Down
hsa-miR-505-5p	14.53138	0.035235	0.003013244	0.001272	Down
hsa-miR-618	15.53962	0.012904	0.001781216	0.000451	Down
hsa-miR-889-3p	10.99614	0.072196	0.006785647	0.002993	Down

Con-EV: EVs from control group; AMI-EV: EVs from AMI group.

TABLE S2. The qPCR primers used in the study.

Species	Name	Primer	Sequence
Human	has-miR-503	Forward	5'-UAGCAGCGGGAACAGUUCUGCAG-3'
		Reverse	5'-GTGCAGGGTCCGAGGT-3'
Mouse	mmu-miR-503	Forward	5'-UAGCAGCGGGAACAGUACUGCAG-3'
		Reverse	5'-GTGCAGGGTCCGAGGT-3'
Mouse	pre-mmu-miR-503	Forward	5'-AGGACTGGAATGGGACCCA-3'
		Reverse	5'-CGAGTAGCTGAAACGCATGC-3'
Mouse	pri-mmu-miR-503-site-1	Forward	5'-CACTATCCACCACACCCTGC-3'
		Reverse	5'-CATGAATTGCTGCTGCAGC-3'
Mouse	pri-mmu-miR-503-site-2	Forward	5'-TTGGAGTATTGCAAGGTTCAA-3'
		Reverse	5'-CCCTGCCCTCCCACCTA-3'
Mouse	pri-mmu-miR-503-site-3	Forward	5'-TAGTCTAGTGAGGGTCACTCTACACAGTC-3'
		Reverse	5'-CACTGCAGTACTGTTCCCGC-3'
Mouse	pri-mmu-miR-503-site-4	Forward	5'-CTTCCACATGGGCACACC-3'
		Reverse	5'-CTTCCACATGGGCACACC-3'
Mouse	U6	Forward	5′-GCTTCGGCAGCACATATACTAAAAT-3′
		Reverse	5′-CGCTTCACGAATTTGCGTGTCAT-3′
Mouse	PGC-1β	Forward	5′-TGCAGCTAAGAGACCATGAGATCCGT-3′
		Reverse	5′-GCTTCTTTAGTTCCCGAGAGCTCATGTC-3′
Mouse	SIRT3	Forward	5′- ATCCCGGACTTCAGATCCCC-3′
		Reverse	5′- CAACATGAAAAAGGGCTTGGG-3′
Mouse	Ndufa1	Forward	5′-CCGGAAGAGAGGTAAAGCCG-3′
		Reverse	5′-ACATCTCCGCACCGTTACTC-3′
Mouse	Ndufa2	Forward	5′-CGCTGTACAGTGTCCCTTCA-3′
		Reverse	5′-CAGTGTTGCGCAGTAAGAGG-3′
Mouse	Ndufa3	Forward	5′-GGCCACACCCTACAACTACC-3′
		Reverse	5′-CAGGCATGTTCCCGTCATCT-3′

Mouse	Ndufb8	Forward	5′-GGAATCGTGTGGACACGTCA-3′
		Reverse	5′-GTACTGCTTCGGACCCACAG-3′
Mouse	Ndufs3	Forward	5′-ATCCTTGGCTGACTTGACGG-3′
		Reverse	5′-CGCAGAGACAGCAGGTTGTA-3′
Mouse	SDHB	Forward	5′-CGCTGCCACACCATCATG-3′
		Reverse	5′-TTTCCGCAATCGCTTTCC-3′
Mouse	Fe-S	Forward	5′-GCTGGGCGCACACTTTGT-3′
		Reverse	5′-CACTGGCCTTGCAGGAAGAA-3′
Mouse	NRF-1	Forward	5′-CTGCCGTAGCAACAGGAAAGA-3′
		Reverse	5′-TCGCAGTTTCCTTAGCAGACG-3′
Mouse	METTL3	Forward	5′-CATCCGTCTTGCCATCTCTAC-3′
		Reverse	5′-GACCTCGCTTTACCTCCAATCA-3′
Mouse	METTL14	Forward	5′-GCATCACTGCGAATGAGAAATG -3′
		Reverse	5′-CAGAACCGCACCAGAGAAATA-3′
Mouse	WTAP	Forward	5′-CACCACTCAAATCCAGTACCTC -3′
		Reverse	5′-AGTCTGTTCCAGTTCACCTTTC-3′
Mouse	HNRNPA2B1	Forward	5′-TGGCTTTGTTACTTTTGATGA-3′
		Reverse	5′-TTCTGTTACCTCTGGGCTCTC-3′
Mouse	β-actin	Forward	5′-GGTCACCCACACTGT GCCCAT-3′
		Reverse	5′-GGATGCCACAGGACT CCATGC-3′
Mouse	METTL3 ChIP	Forward	5′-CTACACAGTCCAAGGGCCTG-3′
		Reverse	5′-GCACCAAACACTCACTGCAG-3′
Mouse	si-METTL3-1	Primer	5′-CTGCACTTCAGACGAATTA-3′
	si-METTL3-2	Primer	5′-GCTACCGTATGGGACATTA-3′
mouse	sh-HNRNPA2B1-1	Primer	5′-GGAACAUCACCUUAGAGAUUACUTT-3′
	sh-HNRNPA2B1-2	Primer	5′-CGCCAAUCAGGUUAGCCUUCUCUTT-3′
Mouse	si-KDM5A-1	Primer	5′-GAAGAAUUCUAGCCAUACA-3′
	si-KDM5A-2	Primer	5′-GGAAAUACCCAGAGAAUGA-3′
Mouse	si-PGC-1β	Primer	5′-CCACCACACCTCCATATAA-3′

Mouse	si-SITR3	Primer	5′-CACCGCTTTCAACAAACCTCCAGGG-3′
Mouse	si-NRF-1	Primer	5′-AGAGCATGATCCTGGAAGA-3′

TABLE S3. Sequences of synthetic miRNA mimics and inhibitor.

Species	Name	Primer	Sequence
Human	hsa-miR-503	Forward	5'-UAGCAGCGGGAACAGUUCUGCAG-3'
		Reverse	5'-AUCGUCGCCCUUGUCAAGACGUC-3'
Mouse	mmu-miR-503	Forward	5'-UAGCAGCGGGAACAGUACUGCAG-3'
		Reverse	5'-AUCGUCGCCCUUGUCAUGACGUC-3'
Mouse	anti-mmu-miR-503	5’-mCmUmGmCmAmGmUmAmCmUmGmUmUmCmCmCm GmCmUmGmCmUmA-3’	

TABLE S4. Primary Antibodies

	Isotype/Manufacturer/Catalog	Concentration
Alix	Rabbit IgG/Abcom/ab186429	1:2000
TSG101	Rabbit IgG/Abcom/ab125011	1:2000
CD63	Rabbit IgG/Abcom/ab134045	1:1000
Calnexin	Rabbit IgG/Abcom/ab133615	1:1000
TnI	Rabbit IgG/Affinity/AF7807	1:400
PGC-1β	Rabbit IgG/Abcom/ab176328	1:5000
SIRT3	Rabbit IgG/Abcom/ab189860	1:1000
NDUFB8	Rabbit IgG/Abcom/ab192878	1:5000
SDHB	Mouse IgG/abcom/ab14714	1:1000
Kac	Rabbit IgG/CST/#9441	1:100 for IP and 1:1000 for WB
PDH	Rabbit IgG/CST/C54G1	1:1000
ATP synthase	Rabbit IgG/Abcom/ab176569	1:1000
METTL3	Rabbit IgG/Abcom/ab195352	1:400 for IF and 1:1000 for WB

HNRNPA2B1	Rabbit IgG/Abcom/ab31645	1:1000
H3K4me3	Mouse IgG/abcom/ab12209	1:200 for IF and 1:1000 for WB
H3K4me2	Rabbit IgG/Abcom/ab32356	1:1000
H3	Rabbit IgG/Abcom/ab1791	1:1000
KDM5A	Rabbit IgG/Abcom/ab194286	1:1000
β-actin	Mouse IgG/Proteintech/66009-1-lg	1:1000
